# Plin2 deletion increases cholesteryl ester lipid droplet content and disturbs cholesterol balance in adrenal cortex

**DOI:** 10.1016/j.jlr.2021.100048

**Published:** 2021-02-11

**Authors:** Yuchuan Li, Prabhat Khanal, Frode Norheim, Marit Hjorth, Thomas Bjellaas, Christian A. Drevon, Jarle Vaage, Alan R. Kimmel, Knut Tomas Dalen

**Affiliations:** 1 Department of Nutrition, Institute of Basic Medical Sciences, Faculty of Medicine, University of Oslo, Oslo, Norway; 2 Faculty of Biosciences and Aquaculture, Nord University, Steinkjer, Norway; 3 VITAS AS, Oslo, Norway; 4 Department of Molecular Medicine, Institute of Basic Medical Sciences, University of Oslo, Oslo, Norway; 5 Institute of Clinical Medicine, University of Oslo, Oslo, Norway; 6 Department of Research and Development, Division of Emergencies and Critical Care, Oslo University Hospital, Oslo, Norway; 7 Laboratory of Cellular and Developmental Biology, National Institute of Diabetes and Digestive and Kidney Diseases, The National Institutes of Health, Bethesda, MD, USA; 8 The Norwegian Transgenic Center, Institute of Basic Medical Sciences, University of Oslo, Oslo, Norway

**Keywords:** Plin1, Plin2, Plin3, cholesteryl ester, lipid droplets, adrenal cortex, cholesterol balance, corticosterone, phosphatidylglycerol, lysophosphatidylcholine, *Abca1*, ATP-binding cassette, subfamily A, member 1, *Abcg1*, ATP-binding cassette, subfamily G, member 1, ACTH, adrenocorticotropic hormone, *Adgre1*, adhesion G protein-coupled receptor E1, ApoE, apolipoprotein E, CE, cholesteryl ester, *Cyp11a1*, cytochrome P450 family 11 subfamily A member 1, DAG, diacylglycerol, *Hmgcr*, 3-hydroxy-3-methylglutaryl-CoA reductase, HSL, hormone sensitive lipase, Lamp, lysosome-associated membrane protein, LC3, microtubule-associated protein 1A/1B-light chain 3, *Ldlr*, low density lipoprotein receptor, LD, lipid droplet, *Lipe*, lipase E (Hsl), LPC, lysophosphatidylcholine, *Nr1h2*, nuclear receptor subfamily 1 group H member 2 (LXRβ), *Nr1h3*, nuclear receptor subfamily 1 group H member 3 (LXRα), OCT, optimal cutting temperature, p62, ubiquitin-binding protein p62, *Pcyt1a*, phosphate cytidylyltransferase 1, choline, alpha isoform, PB, phosphate buffer, PFA, paraformaldehyde, PG, phosphatidylglycerol, *Scarb1*, scavenger receptor class B, member 1, *Soat1*, sterol O-acyltransferase 1, *Srebf1c*, sterol regulatory element binding transcription factor 1 isoform c, *StAR*, steroidogenic acute regulatory protein, TAG, triacylglycerol, *Tbp*, TATA-box binding protein

## Abstract

Cholesteryl esters (CEs) are the water-insoluble transport and storage form of cholesterol. Steroidogenic cells primarily store CEs in cytoplasmic lipid droplet (LD) organelles, as contrasted to the majority of mammalian cell types that predominantly store triacylglycerol (TAG) in LDs. The LD-binding Plin2 binds to both CE- and TAG-rich LDs, and although Plin2 is known to regulate degradation of TAG-rich LDs, its role for regulation of CE-rich LDs is unclear. To investigate the role of Plin2 in the regulation of CE-rich LDs, we performed histological and molecular characterization of adrenal glands from *Plin2*^+/+^ and *Plin2*^−/−^ mice. Adrenal glands of *Plin2*^−/−^ mice had significantly enlarged organ size, increased size and numbers of CE-rich LDs in cortical cells, elevated cellular unesterified cholesterol levels, and increased expression of macrophage markers and genes facilitating reverse cholesterol transport. Despite altered LD storage, mobilization of adrenal LDs and secretion of corticosterone induced by adrenocorticotropic hormone stimulation or starvation were similar in *Plin2*^+/+^ and *Plin2*^−/−^ mice. *Plin2*^−/−^ adrenals accumulated ceroid-like structures rich in multilamellar bodies in the adrenal cortex-medulla boundary, which increased with age, particularly in females. Finally, *Plin2*^−/−^ mice displayed unexpectedly high levels of phosphatidylglycerols, which directly paralleled the accumulation of these ceroid-like structures. Our findings demonstrate an important role of Plin2 for regulation of CE-rich LDs and cellular cholesterol balance in the adrenal cortex.

Steroid hormones, including glucocorticoids, mineralocorticoids, androgens, estrogens, and progestogens, play essential roles in the regulation of metabolism, blood pressure, water and salt balance, inflammation and immune response, as well as sexual development (1). These hormones are synthesized in specialized endocrine steroidogenic cells mainly in the adrenal cortex, testes, and ovaries. In contrast to most cell types that predominantly store triacylglycerol (TAG), steroidogenic cells primarily store cholesteryl esters (CEs) in cytoplasmic lipid droplets (LDs) (2). LDs consist of a core of neutral lipids surrounded by a single layer of phospholipids and a variety of proteins (3). These LD-associated proteins stabilize the LD surface and maintain the organelle structure or participate in cellular processes such as LD trafficking, interactions with other organelles, cell signaling, or catalysis of enzymatic reactions. The LD-associated proteins dynamically regulate growth and degradation of LDs in response to changes in cellular lipid flux and lipid metabolism (4, 5).

The adrenal gland consists of two distinctly different endocrine moieties. The interior medulla consists of chromaffin cells that produce catecholamines like adrenaline and noradrenaline. These hormones are stored intracellularly for rapid release (6). The exterior cortex produces steroid hormones such as mineralocorticoids (aldosterone), glucocorticoids (cortisone and cortisol), and androgens. The steroid hormones cannot be stored in large quantities but are released from the cortical cells as they are synthesized (7). Steroid hormone secretion therefore depends on constant and immediate availability of precursor molecules for their synthesis. Cholesterol affects membrane structure and fluidity in all cell types, and it also serves as an important precursor for the synthesis of steroid hormones (steroidogenesis) in cortical cells (8, 9). In response to pituitary signaling by adrenocorticotropic hormone (ACTH), cholesterol in steroidogenic cells is actively transported into the mitochondria by steroidogenic acute regulatory protein (StAR). Thereafter, different P450 enzymes in the mitochondrial matrix and the endoplasmic reticulum synthesize steroid hormones for immediate secretion (8).

Adrenal cortical cells acquire cholesterol from de novo synthesis, intracellular mobilization from membranes and CE-rich LDs, uptake of external cholesterol via endocytosis of LDL particles, and Scavenger receptor class B type 1 (SR-BI)-mediated selective CE uptake (10). External supply of cholesterol is influenced by dietary intake and does not necessarily correlate with the fluctuating demand for cholesterol needed for steroidogenesis. Thus, a well-functioning adrenal cortex depends on reliable internal storage of cholesterol and a high flexibility to buffer intracellular cholesterol to avoid its overload. Cholesterol balance is partly achieved by buffering in cellular membranes (11), whereas CE-rich LDs are more efficient owing to their rapid sequestration and release of cholesterol by esterification and lipolysis, respectively (2).

Mobilization of CE-rich LDs in adrenal cortical cells occurs via two different pathways, similar to degradation of TAG-rich LDs in other cell types. The lipophagic degradation pathway is a special form of autophagy where LDs are partially or completely engulfed by phagophores, followed by fusion with lysosomes where LD components are degraded by lysosomal acid lipases and other enzymes (12, 13). The second hormonally controlled lipolytic pathway involves enzymatic degradation of the LD core by cytosolic neutral lipases (14). Both pathways promote the release of unesterified cholesterol and fatty acids to the cytoplasm. The main neutral lipase degrading neutral CE in the adrenals is hormone sensitive lipase (HSL) (15). HSL is phosphorylated at serine-660 and serine-565 by protein kinase A (16) following, e.g., ACTH stimulation. Phosphorylation leads to a modest increase in HSL activity and, more importantly, directed recruitment to the LD surface (17) assisted by LD scaffolding proteins of the perilipin (Plin) family (18). This combination of HSL enzyme activation and LD-anchoring facilitating substrate proximity can increase lipolysis by more than 50-fold. Deletion of the *Lipe* gene encoding HSL suppresses nearly all lipolytic activity in the adrenals, resulting in accumulation of CE-rich LDs in cortical cells in its absence (14, 15).

In mammals, the Plin family includes five *Plin* genes (*Plin1-5*), some of which are alternatively spliced, resulting in at least 10 perilipin protein isoforms in mice (19). Variations in their tissue expression, hormone-induced activation by phosphorylation, lipase recruiting abilities, and interactions with the autophagic machinery enable Plins to regulate LD stability differently (20, 21). The roles of individual Plins in the regulation of adrenal LDs are uncharacterized. Cultured murine Y1 adrenal cortical cells are known to express Plin1a, Plin1c, and Plin2 mRNAs (19, 22), with CE-rich LDs coated with both Plin1 and Plin2 proteins (22, 23). In contrast, adipocytes express Plin1a, Plin1b, Plin2, and other Plin mRNAs but store TAG-rich LDs that are coated mainly with Plin1, as translated Plin2 is rapidly ubiquitylated and degraded (24). Of note, TAG-rich and CE-rich LDs tend to recruit Plin1a/b and Plin1c isoforms, respectively (25), whereas the Plin2 protein (26) shows no preference to LDs filled with TAG or CE (25). Expression levels for Plin3-5 are unknown in adrenal cortical cells, as these Plin family members were discovered after the initial investigations.

While dissecting a small colony of old *Plin2*^+/+^ and *Plin2*^−/−^ mice, we noted an unexpected enlargement of the adrenal glands in all examined *Plin2*^−/−^ mice. The purpose of this study was to follow up with detailed examination of storage and regulation of CE-rich LDs in *Plin2*^−/−^ adrenals. We demonstrated that lack of Plin2 caused unexpected alterations in CE storage in adrenal cortical cells and an age-dependent accumulation of multilamellar ceroid-like structures in the cortex-medulla boundary.

## Materials and methods

### Materials

Plasticware was obtained from Costar (Merck, Darmstadt, Germany) and Sarstedt (Nümbrecht, Germany). Materials for real-time quantitative PCR were obtained from Applied Biosystems (Thermo Fisher Scientific, Waltham, MA). Organic solvents for TLC, paraformaldehyde, glutaraldehyde, and Hoechst 33342, were obtained from Sigma-Aldrich (St. Louis, MO). Phalloidin-CF568 conjugate was obtained from Biotium (Fremont, CA). Bodipy 493/503 was obtained from Thermo Fisher Scientific.

Lipids used for TLC were purchased from two sources: 2-oleoyl-1-palmitoyl-sn-glycero-3-phosphocholine (#42773), 2-oleoyl-1-palmitoyl-sn-glycero-3-phosphoethanolamine (#01991), and 2-oleoyl-1-palmitoyl-sn-glycero-3-phospho-L-serine sodium salt (#51581) were obtained from Sigma-Aldrich (St. Louis, MO). Hexadecanoic acid (palmitic, #N-16-A), octadecanoic acid (stearic, #N-18-A), 9-hexadecenoic acid (palmitoleic, #U-40-A), 9-octadecenoic acid (oleic, #U-46-A), 7-octadecenoic acid (vaccenic, #U-48A), 9-12 octadecadienoic acid (linoleic, #U-59-A), trihexadecenoin (palmitolein, #T-215), 13-docosenoic acid (erucic, #U-79-A), dipalmitolein (#D-216), trioctadecenoin (#T-235), diolein (#D-236), diolein 1-3 Isomer (#D-237), monoolein (#M-239), cholesteryl palmitate (#CH-815), cholesteryl palmitoleate (#CH-826), and cholesteryl oleate (#CH-828) were obtained from Nu-Chek Prep Inc. (Elysian, MN).

### Cloning of the Plin2 targeting vector

The Plin2-Flox-Neo construct was generated using recombineering (27). Briefly, a prescreened BAC clone obtained from AB2.2 ES cells (strain: 129S7/SvEvBrd-Hprtb-m2, clone: #bMQ-28H12; Sanger, United Kingdom (28)) was electroporated into Sw102 cells. Thereafter, a 19 kb segment spanning the whole Plin2 locus was inserted with gap repair into a pL253 vector containing a thymidine kinase gene (pL253-Plin2). The two mini cassettes needed to insert LoxP sites were constructed in pPCR-Script (Stratagene). Fragments were digested out with NotI/SalI, purified on agarose gel, and sequentially electroporated (∼200 ng) with the pL253-Plin2 vector (∼5 pg) into Sw102 cells to insert a LoxP site in intron 3 and an FRT-Neo-FRT-LoxP cassette in intron 6, resulting in the final Plin2 targeting vector (Plin2-Flox-Neo, see Supplemental Fig. S1). Primers used for cloning are shown in Supplemental Table S1.

### Generation of the Plin2 floxed and null model

The linearized Plin2-Flox-Neo vector was electroporated into 129/svJEv ES cells. ES cell clones with the desired homologous recombination event were identified by Southern blotting using Turbo blotter (Schleicher & Schuell) followed by hybridization of membranes with [α-^32^P]dCTP (PerkinElmer, Wellesley, MA) radiolabeled probes generated with the Megaprime DNA labelling System (Amersham Biosciences). Targeting efficiency was 10 positive events of 96 screened clones.

Animal use for generation of the floxed *Plin2* model was registered with and approved by the NIDDK Animal Care and Use Committee (plan #K039-LCDB-10) and followed the national Guide for the Care and Use of Laboratory Animals. Positive ES cells were microinjected into blastocysts derived from C57BL/6J females and transferred to pseudopregnant recipients. Chimeric offspring were mated with C57BL/6J mice for germline transmission. The presence of the targeted allele in the agouti-colored offspring was confirmed by PCR and Southern blot hybridization. Floxed *Plin2* mice (*Plin2*^flox-Neo^) were mated with mice expressing MMTV-Cre recombinase to obtain mice with global deletion of Plin2 (*Plin2*^−/−^) mice, which were confirmed for deletion of exons 3, 4, and 5 by PCR, RT-PCR, and Southern blot hybridization (see Supplemental Fig. S1).

### Animal experiments

All experimental use of animals was approved and registered by the Norwegian Animal Research Authority (Mattilsynet, approvals FOTS ids: #6305, #6922, and #10901) and conformed to the ARRIVE guidelines and ethical guidelines in Directive 2010/63/EU of the European Parliament on the protection of animals used for scientific purposes. Mice were housed with a stable light/dark cycle (7 AM–7 PM), with 55 ± 5% relative humidity at 22 ± 2°C, with free access to water and rodent chow (2018 Teklad global 18% protein rodent diets, Envigo, WI). The presence of pathogens was monitored quarterly in accord with FELASA (Federation of European Laboratory Animal Science Associations) guidelines. All animals in these studies were specific pathogen-free according to FELASA recommendations (SPF status). *Plin2*^−/−^ mice were back-crossed into c57bl/6N for >10 generations prior to experiments. *Plin2*^+/+^ and *Plin2*^−/−^ mice from the same breeding colony were euthanized by cervical dislocation at 08–10 AM. Unless stated otherwise, experiments were performed in animals at 15–16 weeks of age. A total of 166 mice were used in these studies. The number of mice used for each experiment is indicated in the figure legends.

#### Chow feeding and fasting

*Plin2*^+/+^ and *Plin2*^−/−^ animals were housed with free access to standard chow or fasted for 24 h. Chow-fed mice remained in their original cages, whereas mice under fasting were transferred in pairs to clean cages equipped with a plastic shelter but no bedding material or eatable items. *Aging studies*: Male and female *Plin2*^+/+^ and *Plin2*^−/−^ mice remained on chow until they were euthanized. *Response to ACTH*: At 9:00 AM, male and female *Plin2*^+/+^ and *Plin2*^−/−^ mice (15 weeks) were injected intraperitoneally with 0.1 μg/g body weight ACTH fragment 1-24 (Cat. # A0298, Sigma-Aldrich, St. Louis, MO) dissolved in 0.9% saline. Blood samples were taken from the lateral saphenous vein with capillary blood collection tubes (#20.1280.100, Sarstedt) before ACTH injection (baseline) and 1 and 2 h after injection. Serum was retrieved by centrifugation of collected blood samples at 10,000 *g* for 4 min at room temperature, then snap frozen in liquid nitrogen, and stored at −80°C until analysis. Corticosterone levels in serum were determined by ELISA (#EIA-4164, DRG Instruments GmbH, Marburg, Germany).

### Collection of adrenal glands

Depending on the downstream analyses, three different procedures were used for the collection of adrenals. For histological studies, the adrenals were dissected together with the surrounding fat capsule and fixed overnight at 4°C with 4% paraformaldehyde (PFA) in 0.1 mol/L phosphate buffer (4% PFA-PB, pH 7.4) prior to storage in 0.4% PFA in 0.1 mol/L phosphate buffer (0.4% PFA-PB, pH 7.4) at 4°C. Visible fat was carefully removed under a stereo microscope prior to measurement of organ weights and embedding for histology. For mRNA analysis, the dissected adrenals were preserved in RNAlater solution (Cat. # AM7020, Thermo Fisher Scientific) and stored at −20°C. The surrounding fat layer was carefully removed under a stereo microscope, and the cleaned adrenals were washed with RNAlater solution prior to RNA isolation. For lipid composition and protein analyses, freshly dissected adrenals were cleared of visible fat in cold PBS under a stereo microscope within 5 min after euthanasia, and samples were snap frozen in liquid nitrogen and transferred to −80°C until analyses.

### Isolation of total RNA

Total RNA was isolated with NucleoSpin RNA kit (MACHEREY-NAGEL, GmbH & Co. KG, Düren, Germany) using a modified protocol to increase RNA yield and purity. Briefly, the adrenals were homogenized in RA1 lysis buffer (350 μl per sample, provided in kit) supplemented with 1% 2-mercaptoethanol (Sigma-Aldrich, Steinheim, Germany) using Precellys 24 tissue homogenizer (Bertin Technologies, Montigny-le-Bretonneux, France). The lysate was subsequently mixed with 350 μl phenol:chloroform:isoamyl alcohol (25:24:1, v/v) (Invitrogen, CA), extracted by vigorous shaking for 20 s, and left on the bench for about 5 min at room temperature. The aqueous and organic phases were separated by centrifugation at 9,000 rpm for 5 min. The upper aqueous phase (∼350 μl) was transferred to a clean tube, mixed with 95 μl high-salt solution (1.5 mol/L NaCl and 0.8 mol/L NaOAc), mixed with 260 μl 96% ethanol and loaded on the silica column for RNA purification according to the manufacturer's instructions. RNA was eluted in 40 μl RNase-free water. The concentration and quality (260/280 ratio) of individual RNA samples were determined on a NanoDrop ND-1000 Spectrophotometer (Thermo Fisher Scientific). Isolated RNA was stored at −80°C.

### Reverse transcription quantitative polymerase chain reaction

#### Reverse transcription of RNA

Total RNA (12.5 ng/μl) was reversely transcribed with random hexamers and the High Capacity cDNA Reverse Transcription Kit (Cat. # 4368814, Thermo Fisher Scientific) on an ep Gradient S Eppendorf Mastercycler (Eppendorf AG, Hamburg, Germany) with the following settings: 25°C for 10 min, 37°C for 120 min, 85°C for 5 min, and 4°C on hold.

#### qPCR with fluorescent intercalating dye

Gene-specific regions were amplified from cDNA (5–10 ng/μl) with assay primers (200 nmol/L each) and Bio-Rad SsoAdvanced™ Universal SYBR® Green Supermix (10 μl reaction, 95°C for 3 min, followed by 40 cycles of 95°C for 10 s and 60°C for 20 s) on the ABI 7900HT system (Thermo Fisher Scientific). Assay primers were designed with Primer-BLAST software (29). Assay primer pairs were designed to span a large intron with binding sites on adjacent exons with similar melting points (Tm = 60) and amplicon sizes ranging from 70 to 120 nucleotides. SDS 2.3 software was used for the experimental setup. Assay sequences are listed in Supplemental Table S2.

#### Quantification of reverse transcription quantitative polymerase chain reaction data

Expression analysis was performed with RQ Manager 1.2 (Applied Biosystems, Life Technologies) using the relative quantification method (ΔCq method). Tata-binding protein (*Tbp*) mRNA was verified to be stably expressed among groups and treatments and was used as a reference gene in all experiments. Gene expression data are presented as gene expression levels relative to expression of *Tbp* (2^−ΔCq^). This normalization method gives indications of gene expression levels relative to other expressed genes (weakly or highly expressed).

### Immunoblotting

Adrenals were lysed in RIPA buffer containing complete proteinase inhibitor cocktail (Cat. #11836170001, Roche, Basel, Switzerland) and phosphatase inhibitors (Cat. #P0044, Sigma-Aldrich, St. Louis, MO). Samples were homogenized using glass beads and a Precellys 24 tissue homogenizer (Bertin Instruments, Montigny-le-Bretonneux, France) and sonicated on a Bioruptor® Plus device (Diagenode, Liege, Belgium). Lysates were diluted in Laemmli buffer; proteins were separated on Criterion™ TGX™ 4–20% gels (Bio-Rad, Hercules, CA) and transferred to nitrocellulose membranes using the Trans-Blot Turbo transfer system and RTA transfer kit (Bio-Rad). Membranes were stained with Ponceau S to quantify total protein loaded in each lane to normalize chemiluminescence signals. Membranes were blocked in Tris buffered saline containing 0.1% Tween-20 (TBS-T) and 5% BSA and incubated overnight with primary antibodies in TBS-T containing 2.5% BSA. After washing, the membranes were incubated with appropriate HRP-conjugated secondary antibodies. The following primary antibodies were used: rabbit anti-Gapdh (#sc-25778, Santa-Cruz Biotechnology, Dallas, TX), guinea pig anti-Plin2 (#GP-40, Progen, Heidelberg, Germany), guinea pig anti-Plin1 (#20R-PP004, Fitzgerald, Acton, MA), rabbit anti-HSL (#4107, Cell Signaling, Danvers, MA), rabbit anti-phospho-HSL(Ser-p565) (#4137, Cell Signaling), rabbit anti-Lamp1 (#3243, Cell Signaling), rabbit anti-LC3B (#2775, Cell Signaling), rabbit anti-SQSTM1/p62 (#5114, Cell Signaling), rabbit anti-Lamp2 (#ab13524, AbCam, Cambridge, United Kingdom), and rabbit anti-Plin3 generated by the Londos laboratory (30). The following secondary antibodies were used: Goat anti-rabbit IgG (#111-035-144), donkey anti-guinea pig IgG (H+L) (#706-035-148, Jackson ImmunoResearch, West Grove, PA), goat anti-rat IgG (H+L) (#AP136P, Sigma-Aldrich, Steinheim, Germany). Chemiluminescence detection was done on the ChemiDoc™ Touch Imaging System (Bio-Rad). Band intensities were quantified using ImageJ v1.52 software (NIH, Bethesda, MD), with protein abundance given relative to total protein levels in the corresponding lane (Gapdh signals are shown to visualize consistent protein loading).

### Tissue embedding, section preparation, staining, and microscopy

Adrenals stored in 0.4% PFA-PB were cryoprotected with a gradient of sucrose-PB solutions (sucrose dissolved in 0.1 mol/L phosphate buffer) at 4°C. The adrenals were first immersed in 10% sucrose-PB for 1 h, then 20% sucrose-PB for 1 h, and finally 30% sucrose-PB overnight. The next day, the tissue was washed quickly with a 1:1 mix of OCT embedding matrix (KMA-0100-00A, CellPath, Newtown, United Kingdom) and PB, followed by a second wash in OCT. Tissues were subsequently placed in embedding molds (E4140-1EA, Sigma-Aldrich, Steinheim, Germany) and covered with fresh OCT. Special care was taken to make sure all the adrenals had similar orientation in the OCT blocks. Finally, the samples in molds were frozen in cold N_2_ vapor by placing the mold 3–5 mm above the surface of liquid nitrogen. Sections with 20 μm thickness were prepared from OCT blocks with a Leica CM3050S cryostat at −20°C, and individual sections were transferred to PB containing 0.02% NaN_3_ and stored in the buffer at 4°C for maximum 2 weeks before use.

Sections were washed once in PB and stained for 25 min while floating in triple-staining solution with gentle agitation (using a 24-well plate, ∼800 μl/well). The triple-staining solution consisted of PB containing 1 μmol/L Bodipy 493/503 (to stain LDs), 5 μmol/L Hoechst-33342 (to stain nuclei), and 1 U/ml CF568 conjugated Phalloidin (to stain cell skeleton). In some experiments, not all of the above stains were used. After staining, sections were washed three times in PB, then mounted on SuperFrost Plus microscope slides (#406/0179/00, VWR, Radnor, PA) with ProLong® Diamond Antifade Mountant (#P36965, Thermo Fisher Scientific) and sealed with glass coverslips (#631-0653, VWR). The mounted sections were allowed to harden overnight at room temperature and stored in the dark under 4°C. All steps from sectioning until imaging were completed within 2 weeks.

#### Whole section scans

Whole sections were imaged to reveal the overall organ structure and distribution of LDs. Bright-field images of adrenal sections were taken under a 20× objective with an Axio Scan Z1 system (Zeiss). Nucleus staining was excited at 330–375 nm and fluorescence detected at 430–470 nm. LD staining was excited at 453–485 nm and fluorescence detected at 507–546 nm. Cell skeleton staining was excited with 540–557 nm and fluorescence detected at 578–640 nm. Autofluorescent signals were excited at 330–375 nm, with emitted signals detected over a broad specter of 430–640 nm.

#### Confocal microscopy

High-resolution confocal images were taken under a 40× oil immersion objective mounted on an LSM 710 confocal microscope (Zeiss). Nucleus staining was excited with a 405 nm laser and fluorescence detected at 414–465 nm. The LD staining was excited with a 488 nm laser and fluorescence detected at 497–545 nm. The cell skeleton staining was excited with a 561 nm laser and fluorescence detected at 563–632 nm. Laser and detection settings were optimized for each channel with a representative section, and the same settings were used for the remaining sections. Autofluorescence was excited with the 405 nm laser, and the emitted fluorescence was detected at 415–465, 495–545, and 565–615 nm.

### Lipid droplet quantification with ImageJ

To give a consistent approximation of adrenal size in sections, the left adrenals were sectioned through their centers and along the longest axis. Sections with a cut plane at the center showing a clear and large medulla area were used for quantification. Confocal pictures (10 from each section with no overlaps) were taken from the middle zone of the adrenal cortex (*zona fasciculata*). LD and nuclei signals from the same image were analyzed separately using ImageJ v1.52 software (NIH) to quantify the number of nuclei and the size and numbers of LDs. Settings for signal enhancements and thresholds were adjusted for a representative picture, and identical settings were used for all images from the same experiment. To validate the quantification procedure, 10 different images were taken from each of three adrenal sections from the same adrenal, and the procedure was repeated in four mice. Experimental variance was found to be lower between sections than between individual images from the same section. Based on these results, we concluded that a high number of images taken from one carefully picked representative section per animal gave the best quantitative data.

### Tissue preparation for transmission electron microscopy

Adrenals were dissected, and the surrounding fat capsules were removed prior to overnight fixation at 4°C in EM fixative (1% paraformaldehyde, 2.5% glutaraldehyde, both of EM grade purity, solved in PB, pH 7.4). Samples were post fixed in 1% OsO_4_ dissolved in PB with moderate shaking for 1–2 h at room temperature, followed by three rinses in PB. Gradient dehydration was performed by sequential immersion for 15 min (each step) in 50, 70, 80, and 96% ethanol and finally in 100% ethanol for 3 × 20 min. Traces of water were removed by 2 × 5 min of immersion in propylene oxide. Prior to embedding, samples were infiltrated with Durcupan mixture (Fluka, Sigma-Aldrich Chemie GmbH, Steinheim, Germany) at 56°C for 30 min, then transferred to freshly prepared Durcupan mixture and incubated at room temperature overnight. On the next day, samples were placed on the bottom of gelatin capsules oriented with the “flat” side facing downward, then immersed in fresh Durcupan mixture, and left to polymerize at 56°C for 48 h.

Ultrathin sections (thickness = 80 nm) were prepared with an ultramicrotome (Leica, Vienna, Austria). Sections were mounted on single-hole grids supported with carbon-coated Formvar films (31), then contrast stained in 1% uranyl acetate water solution followed by 0.3% lead citrate (1 min each). Images were obtained with a Tecnai G2 electron microscope (FEI Company, OR).

### Lipid extraction and thin-layer chromatography

Lipid extracts from the adrenals dissected free from the fat capsule were used for thin-layer chromatography (TLC). A microtube containing 10–15 glass beads (1 mm) was precooled on dry ice, and one frozen adrenal was added. For homogenization, 400 μl cold PBS containing 1× complete proteinase inhibitor cocktail (Cat. #11836170001, Roche, Basel, Switzerland) was added and samples were rapidly shaken (5,000 rpm, 30 s ×2) with a Precellys 24 tissue homogenizer (Bertin Technologies, Montigny-le-Bretonneux, France). A fraction of the homogenate (100 μl) was transferred to a separate Eppendorf tube and stored at −80°C until measurement of the protein concentration with bicinchoninic acid assay (#23227, Pierce Biotechnology; Rockford, IL). The remaining homogenate (300 μl) was extracted with 2× volume (600 μl) of lipid extraction solvent [chloroform: heptane: methanol (4:3:2 v/v)], and the tubes were strongly vortexed for 2 × 15 s and left at room temperature for >15 min. The whole liquid phase was then transferred to a new Eppendorf tube and centrifuged at 2,000 *g* for 5 min. The lower organic phase was subsequently transferred to a clean glass tube, dried under N_2_ gas for ∼10 min at 37°C, and stored at −20°C in a sealed tube filled with argon to prevent lipid oxidation. The dried extracted lipids were redissolved in chloroform:methanol (2:1 v/v) to a final concentration equal to 1 μg protein/μl homogenate.

TLC was performed on Silica gel 60 plates (#105748, Merck Millipore, Billerica, MA) as described previously (32). Briefly, to analyze the major lipid species, extracted lipids (normalized against 20 μg adrenal weight for all major lipids, or 2.5 μg protein for cholesterol quantification) and the lipid standard mix [equal weights of TAG, diacylglycerol (DAG), monoacylglycerol, phospholipid, FFA, CE, and cholesterol] were developed in heptane:diethyl ether:acetic acid (55:45:1 v/v). To analyze phospholipid classes, the extracted lipids (normalized against 100 μg adrenal weight) and lipid standard mix [equal weights of phosphatidylcholine (PC), phosphatidylethanolamine (PE), and phosphatidylserine (PS)] were first developed in dichloromethane:ethyl acetate:acetone (80:16:4 v/v) to remove neutral lipids, then in chloroform:ethyl acetate:acetone:isopropanol:ethanol:methanol:water:acetic acid (30:6:6:6:16:28:6:2 v/v) to separate different phospholipids. After separation, the TLC plates were dried for 10 min at 40°C and soaked for 1 min in copper sulfate-phosphoric acid solution [10% CuSO_4_ × 5H_2_O (w/v) and 8% H_3_PO_4_ (v/v) in water]. For visualization of cholesterol and CE, plates were heated for 10 min at 60°C. For visualization of all major lipid species, plates were heated for an additional 5 min at 150°C. The TLC plates were scanned with an image scanner (Epson Perfection V700, Epson, Japan), and band intensities were measured with ImageJ v1.52 software (NIH). The content of cholesterol and CE was calculated based on cholesterol and cholesteryl-oleate standard curves.

### Lipids analysis

Adrenals dissected free from the fat capsule were used for lipids analyses using high-performance liquid chromatography (HPLC) coupled to time of flight-mass spectrometry (TOF-MS). This HPLC-qTOF/MS platform allows determination of separate lipid species such as glycerolipids, glycerophospholipids, sphingolipids, free fatty acids (FFAs), and CE. Whole adrenal tissue samples were transferred to HPLC vials, and 800 μl of isopropanol (LC-MS grade, Fischer Scientific, Waltham, MA) containing internal standards at a concentration of about 1 μg/ml was added. The samples were subsequently homogenized prior to centrifugation at 4,000 rpm at 10°C for 10 min. The supernatant was transferred to an HPLC vial and subsequently analyzed by a 1260 Agilent chromatographic system including an auto-sampler, a binary pump, and a TCC column heater unit coupled to a TOF-MS with Agilent JetStream ionization module for enhanced sensitivity. The system was operated in both positive and negative ionization modes. To obtain high-resolution chromatographic separation of the lipids, an EVO C18 Kinetex analytical column (3.0 × 150 mm, 2.6 μm) was used with a flow rate of 0.7 ml/min. The eluting mobile phase was generated using A (water:isopropanol:acetonitrile, 47.5:35.0:17.5 v/v, 10 mmol/L ammonium formate) and B (water:isopropanol:acetonitrile, 5:70:25 v/v, 10 mmol/L ammonium formate) mixed as a gradient as follows: 0 min (100% B), 1.9 min (100% B), 2.0 min (100% A), 5.0 min (100% A), 17 min (40% B), 38 min (85% B), 38.1 min (100% B). Injection was delayed 5 min to allow optimal injection conditions, and the injected volume was 4 μl (positive mode) and 9 μl (negative mode). In total, 204 specific lipids within these classes were identified in more than 50% of the samples. Detected lipid signals were normalized against adrenal weights and internal standards. Internal standards used were PC-28:0, PE-28:0, PG-30:0, PA-28:0, PS-28:0, SPM-35:1, Cer-35:1, HexCer-35:1, DAG-30:0, TAG-39:0, CE-19:0, FFA-17:1, and LPC-17:1 (Sigma-Aldrich, St. Louis, MO).

### Statistics

All data were analyzed with Prism 5 (Graphpad Software, CA). ANOVA or two-tailed Student's *t*-test was used to analyze all the data; *P* < 0.05 is defined as significant. Data in graphs are shown as means ± SD or means ± SEM.

## Results

### *Plin2*^−/−^ mice have enlarged adrenal glands with increased lipid content in cortical cells

While characterizing Plin2 function in muscle cultures and heart tissue (32, 33), we observed that *Plin2*^−/−^ mice exhibited an unexpected enlargement of adrenal glands in comparison with *Plin2*^+/+^ controls. So far, phenotypic characterization of our *Plin2*^−/−^ model (see Supplemental Fig. S1) has been concentrated on examining turnover of TAG-rich LDs in different cell types and organs. The adrenal glands store mostly CE-rich LDs (2), and this observation suggested a role for Plin2 in metabolism of CE-rich LDs.

**Fig. 1:**
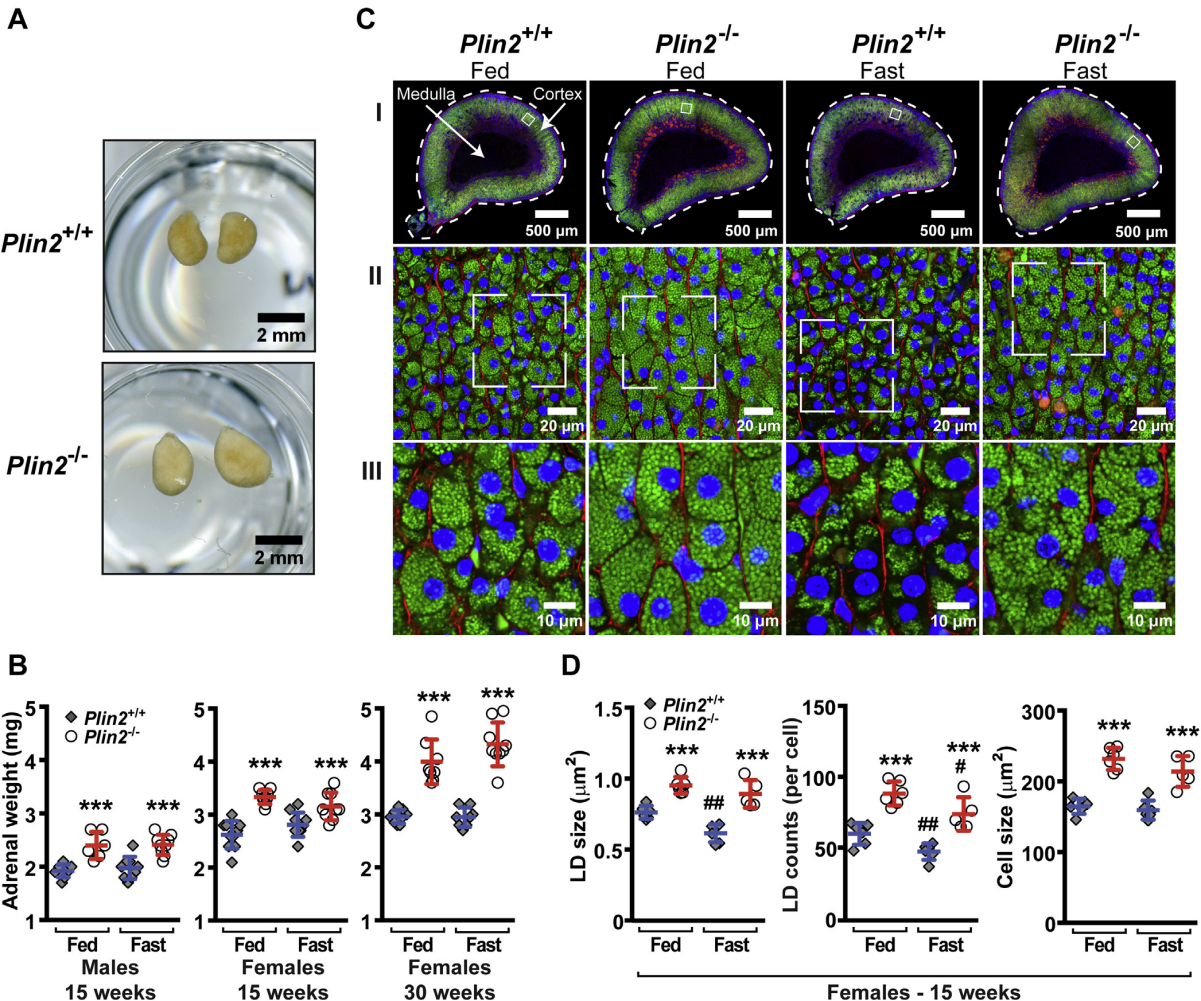
Adrenal glands and lipid droplets are enlarged in *Plin2*^−/−^ mice. *Plin2*^+/+^ and *Plin2*^−/−^ mice were housed with ad libitum access to chow diet (Fed) or were fasted for 24 h (Fast) prior to euthanasia at 8–10 AM. Adrenal glands were dissected out, and the surrounding fat capsule was removed prior to analysis. A: Representative pictures of adrenal glands (pairs) from 30-week-old female *Plin2*^+/+^ and *Plin2*^−/−^ mice. B: Adrenal weights of 15-week-old males (n = 6–9), 15-week-old females (n = 9–10), and 30-week-old females (n = 8–9) presented as means ± SD. Weights are presented as the average weight of the adrenal pair dissected from each mouse. C: Representative pictures of adrenals from fed and fasted 15-week-old female *Plin2*^*+/+*^ and *Plin2*^*−/−*^ mice. Cryosections (20 μm thick) were stained to visualize LDs (Bodipy 493/503, green), nuclei (Hoechst 33342, blue), and plasma membrane-located F-actin (Phalloidin-CF568, red). I: Whole section scans at the center planes of adrenals. II: Confocal images of the central layer of the adrenal cortex (zona fasciculata) taken with a 40× objective. III: Pictures zoomed in from II to visualize individual LDs. Scale bars: 500, 20, and 10 μm. D: Quantification of average LD size, LD number per cell, and cortical cell size based on confocal images. Results are presented as means ± SD (n = 5–6, with 10 images analyzed for each animal). ∗∗∗*P* < 0.001 indicates difference between *Plin2*^*+/+*^ and *Plin2*^*−/−*^ mice; ^#^*P* < 0.05, ^##^*P* < 0.01 indicates difference between fed and fasted mice of the same genotype. LD, lipid droplet.

Further examinations of the adrenals confirmed that the adrenals were enlarged in age-matched 30-week-old female *Plin2*^−/−^ mice compared with *Plin2*^+/+^ mice (Fig. 1A). To determine if mass enlargement of the adrenals was sex dependent or affected by fasting, male and female *Plin2*^+/+^ and *Plin2*^−/−^ mice were studied in the fed state or after 24 h of fasting. Total body weight and liver, heart, and epididymal adipose tissue organ weights were essentially similar in *Plin2*^+/+^ and *Plin2*^−/−^ animals (Table 1). To rule out the possibility that the observed difference in organ size was caused by alterations of the fat capsule surrounding the adrenals, we carefully removed all visible fat before weighing the adrenals. As has been reported previously (34), adrenals were larger in females than in males. However, adrenal mass was enlarged 20–30% in both male and female *Plin2*^−/−^ mice at 15 weeks compared with *Plin2*^+/+^ mice. The increased adrenal mass progressed with age, when comparing 15- and 30-week-old female *Plin2*^−/−^ mice (Fig. 1B). Adrenals from *Plin2*^−/−^ mice also seemed paler in color compared with those from *Plin2*^+/+^ mice (Fig. 1A). Fasting for 24 h, which mobilizes stored CE, had no detectable effect on adrenal weights. This can be explained by the fact that a large fraction of the adrenal gland (the medulla) does not store CE and that CE stored in cortical cells is only partly mobilized after 24 h of fasting, which may mask a significant organ weight change.

**Table 1:** Body weights and organ weights of *Plin2*^+/+^ and *Plin2*^−/−^ mice (15 and 30 weeks)

Age, Sex and Organs	Fed		Fasted (24 h)	
	*Plin2*^+/+^	*Plin2*^−/−^	*Plin2*^+/+^	*Plin2*^−/−^
15 weeks, males				
Body weight (g)	28.0 ± 1.4	28.0 ± 2.1	22.7 ± 2.1^b^	24.5 ± 2.4^b^
Liver (g)	1.46 ± 0.09	1.36 ± 0.16	0.96 ± 0.09^b^	0.97 ± 0.12^b^
Heart (g)	0.128 ± 0.01	0.129 ± 0.008	0.118 ± 0.009^b^	0.132 ± 0.015^a^
WAT epididymal (g)	0.25 ± 0.05	0.21 ± 0.05	0.14 ± 0.11^b^	0.16 ± 0.07
15 weeks, females				
Body weight (g)	21.5 ± 0.9	22.4 ± 1.2	18.0 ± 1.1^b^	18.7 ± 1.4^b^
Liver (g)	0.92 ± 0.08	1.02 ± 0.09^a^	0.81 ± 0.07^b^	0.76 ± 0.09^b^
Heart (g)	0.096 ± 0.008	0.096 ± 0.004	0.091 ± 0.007	0.092 ± 0.008
WAT epididymal (g)	0.21 ± 0.10	0.18 ± 0.08	0.09 ± 0.05^b^	0.11 ± 0.06
30 weeks, females				
Body weight (g)	25.7 ± 1.4	25.1 ± 2.3	21.8 ± 3.6^b^	20.9 ± 1.1^b^

Data are presented as means ± SD, n = 9–10.

a *P* < 0.05 indicates difference between *Plin2*^+/+^ and *Plin2*^−/−^ mice.

b *P* < 0.05 indicates difference between fed and fasted mice of the same genotype.

To determine if alteration in LDs could explain the increased adrenal size in *Plin2*^−/−^ mice, we examined the adrenal LD content and histological structures in 15-week-old female *Plin2*^+/+^ and *Plin2*^−/−^ mice fed *ad libitum* or fasted for 24 h. Adrenal glands were sectioned through their centers to give a consistent approximation of organ size and triple-stained to visualize LDs (green), nuclei (blue), and cell membranes (red). Cross-sectional areas were on average enlarged for *Plin2*^−/−^ adrenals compared with *Plin2*^+/+^ adrenals (Fig. 1C, I), consistent with enlarged organ size and increased organ weight. Staining of the whole sections indicated that LDs accumulated mainly in the steroid hormone-producing outer cortex layer, with no LDs detectable in the central catecholamine-producing medulla. LD staining seemed reduced in *Plin2*^+/+^ adrenals upon fasting, whereas this was less evident in *Plin2*^−/−^ adrenals. To accurately quantify LD differences in adrenals of the two genotypes, we imaged the central layer cells of the adrenal cortex by confocal microscopy (Fig. 1C, II and III) and quantified LD size, LD numbers per cell, and cell size (Fig. 1D). In the fed state, the average LD size increased approximately 25% in *Plin2*^−/−^ adrenal cortical cells and LD numbers per cell increased approximately 50%. These data predict a doubling of stored LD mass per cell in *Plin2*^−/−^ adrenals compared with *Plin2*^+/+^, matching the increased size of these LD-rich cells. LD size and numbers decreased after 24 h of fasting, in agreement with the notion that fasting induces utilization of the stored CE for steroid hormone production and secretion. The mass of LDs (based on LD size and numbers) in *Plin2*^−/−^ adrenals after 24 h of fasting remained higher than for unfasted *Plin2*^+/+^ mice, indicating an enlarged reservoir for CE in *Plin2*^−/−^ adrenals compared with *Plin2*^+/+^ adrenals.

### *Plin2*^−/−^ adrenals have elevated levels of unesterified cholesterol and cholesteryl esters

**Fig. 2:**
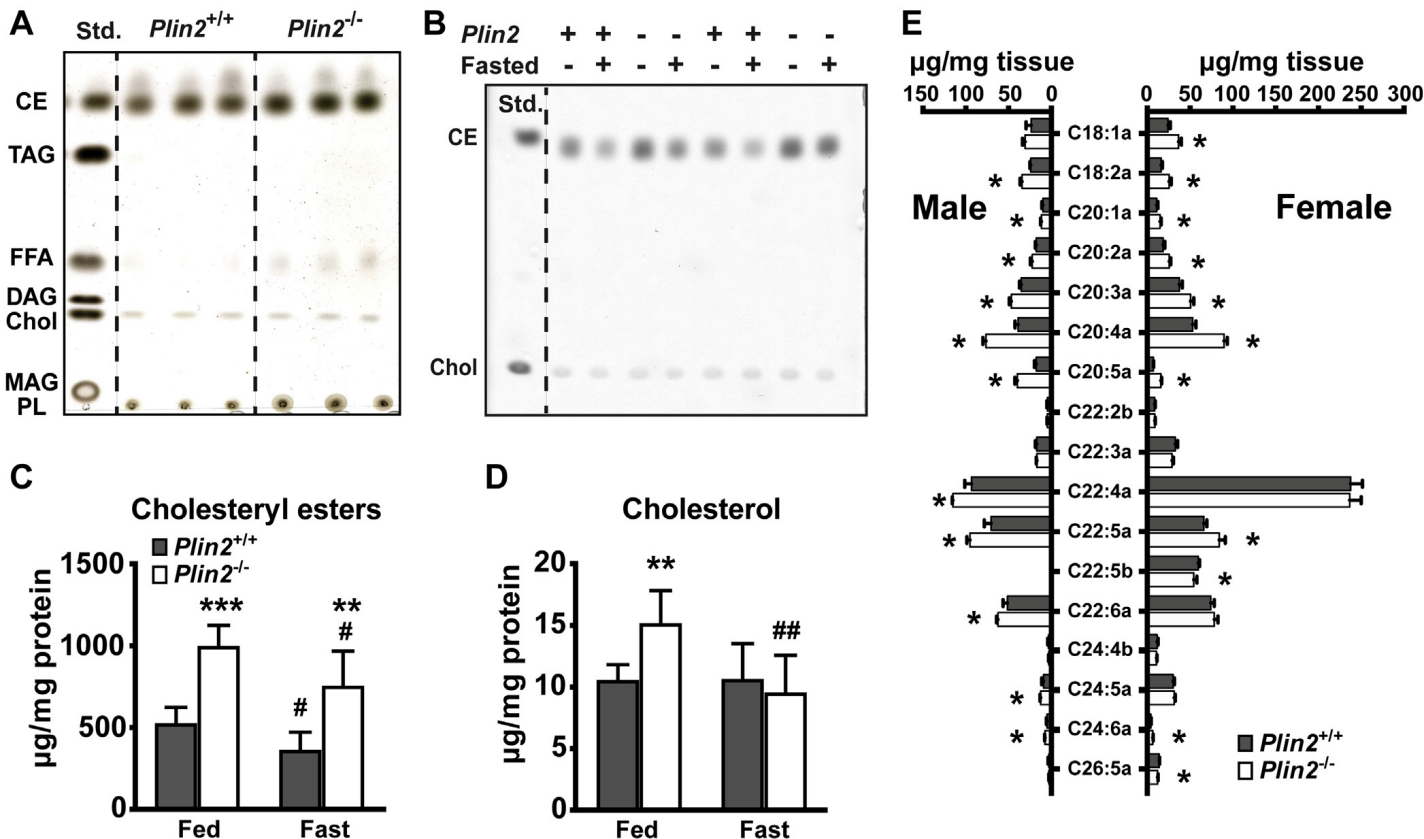
Cholesterol and cholesteryl esters are increased in *Plin2*^−/−^ adrenals. Adrenals collected from fed or 24 h fasted *Plin2*^+/+^ and *Plin2*^−/−^ mice were dissected free from the surrounding fat capsule and analyzed with thin layer chromatography (TLC) or HPLC-qTOF/MS. A: Major neutral lipid species in adrenals of chow-fed 50-week-old female *Plin2*^+/+^ and *Plin2*^−/−^ mice. Lipids normalized against adrenal weight (20 μg) were applied on the TLC plate and developed in heptane:diethyl ether:acetic acid (55:45:1, v/v) to separate the major lipid species. Lipids were visualized with copper sulfate-phosphate solution followed by heating to 150°C (n = 3). B: Cholesteryl ester (CE) and cholesterol content in adrenals of 15-week-old male *Plin2*^+/+^ and *Plin2*^−/−^ mice. Lipids were normalized against protein content (2.5 μg) and separated by TLC. CE and cholesterol were visualized with copper sulfate-phosphate solution followed by heating to 60°C. One representative of three TLC plates is shown. C: Quantification of cholesteryl ester content relative to protein content based on TLC in B (n = 6). D: Quantification of unesterified cholesterol content relative to protein content based on TLC in B (n = 6). E: CE species normalized against adrenal weight of 15-week-old chow-fed female and male *Plin2*^+/+^ and *Plin2*^−/−^ mice analyzed with HPLC-qTOF/MS (n = 3). Results in C-E are presented as means ± SD (∗*P* < 0.05, ∗∗*P* < 0.01, ∗∗∗*P* < 0.001 indicate difference between *Plin2*^*+/+*^ and *Plin2*^*−/−*^ mice; ^#^*P* < 0.05, ^##^*P* < 0.01 indicate difference between fed and fasted mice of the same genotype). CE, cholesteryl ester; Chol, cholesterol; DAG, diacylglycerol; MAG, monoacylglycerol; PL, phospholipid. Std., standard; TAG, triacylglycerol.

Since adrenal LD content was altered in the lack of Plin2, we extracted lipids from *Plin2*^+/+^ and *Plin2*^−/−^ adrenals to determine changes in lipid content. Samples were normalized against adrenal weight prior to separation of the major neutral lipid species with TLC to compare the migration pattern against lipid standards of known concentrations. In the adrenals of 50-week-old females, the major neutral lipids were CE and unesterified cholesterol, without visible levels of TAG, DAG, or monoacylglycerol (Fig. 2A). We also noted indications of increased levels of FFAs in *Plin2*^−/−^ adrenals. Although the relative levels of neutral lipids seemed elevated in *Plin2*^−/−^ adrenals, the classes of stored lipids were unchanged.

To quantify the levels of CE and unesterified cholesterol in cortical cells, lipids extracted from the whole adrenal of 15-week-old males were normalized against protein content and separated with TLC (Fig. 2B). The CE content doubled in *Plin2*^−/−^ adrenals compared with *Plin2*^+/+^ adrenals (Fig. 2C), and the content of unesterified cholesterol increased ∼50% in the fed state (Fig. 2D). Fasting reduced CE content by ∼25% in both *Plin2*^+/+^ and *Plin2*^−/−^ adrenals. Although unesterified cholesterol levels did not change with fasting in *Plin2*^+/+^ adrenals, unesterified cholesterol in *Plin2*^−/−^ adrenals decreased to levels found in *Plin2*^+/+^ adrenals after fasting. Apparently, both *Plin2*^+/+^ and *Plin2*^−/−^ adrenals responded to fasting by degrading CE-rich LDs and mobilized CE with comparable efficiency during the tested time frame.

To compare individual lipid species accumulated in *Plin2*^+/+^ and *Plin2*^−/−^ adrenals, lipids extracted from the adrenals of 15-week-old chow-fed male and female mice were normalized against adrenal organ weight and analyzed with HPLC-qTOF/MS. Normalization against organ weight displays relative changes in individual lipid species in *Plin2*^+/+^ and *Plin2*^−/−^ adrenals. The main species of adrenal CEs were esterified with unsaturated C18 to C22 FAs, such as C18:1, C20:3, C20:4, C22:4, C22:5, and C22:6 (Fig. 2E and Supplemental Fig. S2). CE species that increased in *Plin2*^−/−^ adrenals compared with *Plin2*^+/+^ adrenals consisted mainly of unsaturated C18 and C20 FAs. TAG species were consistently reduced in *Plin2*^−/−^ adrenals compared with *Plin2*^+/+^, although levels were low (Supplemental Fig. S2). Certain phosphatidylglycerol (PG) species were higher in *Plin2*^−/−^ adrenals, whereas lysophosphatidylcholine (LPC) species were unchanged in males but elevated in the adrenals of *Plin2*^−/−^ females. Cardiolipin species were reduced in *Plin2*^−/−^ adrenals. The other measured lipid species remained relatively unaltered.

### Increased levels of Plin3 may compensate for loss of Plin2 in *Plin2*^−/−^ adrenals

**Fig. 3:**
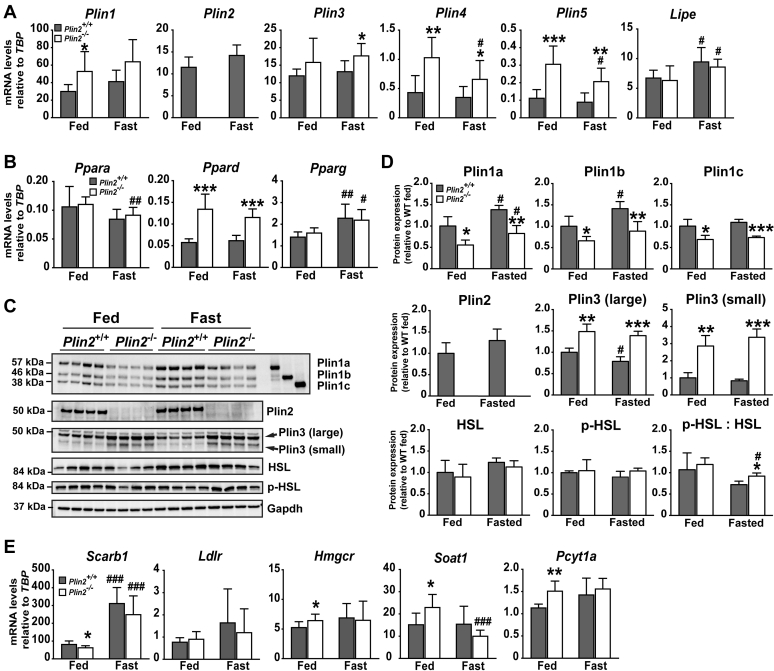
Expression of lipid droplet and cholesterol uptake-related genes in adrenals of *Plin2*^+/+^ and *Plin2*^−/−^ mice. Female *Plin2*^+/+^ and *Plin2*^−/−^ mice at 15 weeks of age had ad libitum access to chow diet (Fed) or were fasted for 24 h (Fast). Mice were euthanized at 8–10 AM. Adrenals were dissected free from the surrounding fat capsule prior to analyses. A: Expression of *Plin1-5* and *Lipe* mRNAs relative to the expression of *Tbp* (n = 8). B: Expression of *Ppars* mRNAs relative to the expression of *Tbp* (n = 8). C: Protein content of Plin1-3, HSL, and HSL phosphorylated at Ser565 (p-HSL). Gapdh is shown to illustrate protein loading per lane. D: Protein content relative to levels measured for fed *Plin2*^+/+^ adrenals by quantification of immunoblots in C (n = 4). E: Expression of *Scarb1*, *Ldlr*, *Hmgcr*, *Soat1*, and *Pcyt1a* mRNAs relative to the expression of *Tbp*. Gene expression data and quantified immunoblots are presented as means ± SD (∗*P* < 0.05, ∗∗*P* < 0.01, ∗∗∗*P* < 0.001 indicate difference between *Plin2*^+/+^ and *Plin2*^−/−^ mice; ^#^*P* < 0.05, ^##^*P* < 0.01, ^###^*P* < 0.001 indicate difference between fed and fasted mice of the same genotype). Gapdh, glyceraldehyde 3-phosphate dehydrogenase; Hmgcr, 3-hydroxy-3-methylglutaryl-CoA reductase; HSL, hormone sensitive lipase; Ldlr, low density lipoprotein receptor; Lipe, lipase E (HSL); Pcyt1a, phosphate cytidylyltransferase 1, choline, alpha isoform; p-HSL, phosphorylated HSL; Ppara, peroxisome proliferator activated receptor alpha (PPARα); Ppard, peroxisome proliferator activated receptor delta (PPARδ); Pparg, peroxisome proliferator activated receptor gamma (PPARγ); Scarb1, scavenger receptor class B, member 1; Soat1, sterol O-acyltransferase 1; Srebf1c, sterol regulatory element binding transcription factor 1 isoform c (SREBP-1C); Tbp, TATA-box binding protein.

Accumulation of CE in *Plin2*^−/−^ adrenal cortical cells may occur owing to altered Plin coating on the LD surface, with potential effects on lipolytic rates and intracellular levels of lipid species activating transcriptional signaling (32). First, we analyzed mRNA expression of the *Plin* family, encoding proteins that regulate lipolytic rate (21), and *Lipe*, encoding the principal neutral lipase degrading neutral CE in adrenals (15). All five *Plin* mRNAs were detected in adrenals, but they could be grouped into three classes based on their expression levels (Fig. 3A). *Plin1* mRNA was the most abundantly expressed and somewhat upregulated in fed *Plin2*^−/−^ adrenals. *Plin2* and *Plin3* mRNAs were highly expressed, with modest upregulation of *Plin3* in fasted *Plin2*^−/−^ adrenals as compared with fasted *Plin2*^+/+^ (Fig. 3A). *Plin4* and *Plin5* mRNAs were expressed at low levels but were elevated about 2-fold in *Plin2*^−/−^ adrenals compared with *Plin2*^+/+^. Fasting had no effect on *Plin1-3* mRNA levels, whereas *Plin4* and *Plin5* mRNA levels were modestly reduced by fasting in *Plin2*^−/−^ adrenals toward levels observed for *Plin2*^+/+^. Expression of *Lipe* mRNA encoding for HSL was unaltered in expression upon loss of Plin2 but was slightly elevated by fasting.

Lipolysis products of CE, such as FFAs or their derivatives, activate peroxisome proliferator-activated receptors (*Ppars*) (35, 36). Because we observed increased expression of the direct *Ppar* target genes *Plin1*, *Plin4*, and *Plin5* (37, 38, 39, 40) in *Plin2*^−/−^ adrenals, we also measured the expression levels of this transcription factor family. Expression of *Ppard* was doubled in *Plin2*^−/−^ adrenals in fed and fasted conditions (Fig. 3B). *Plin2* deletion had no effect on the expression of *Ppara* and *Pparg* mRNAs, but *Pparg* mRNA increased slightly by fasting. These results suggest that PPARδ activity wase elevated in *Plin2*^−/−^ compared with *Plin2*^+/+^ adrenals.

Protein levels of Plins are often inconsistent with their relative mRNA levels, owing to differences in translation rates and posttranslational stability (24, 41). We next examined adrenal protein levels of the highly transcribed *Plin1-3* with immunoblotting. The adrenals expressed nearly similar levels of the Plin1a-1c isoforms (Fig. 3C, D), partly different from Y1 adrenal cortical cultured cells that express Plin1a and Plin1c (22). Despite increased *Plin1* mRNA in *Plin2*^−/−^ adrenals (Fig. 3A), Plin1a-c protein levels decreased about 50% in *Plin2*^−/−^ compared with *Plin2*^+/+^ (Fig. 3C). Plin2 protein was unaffected by fasting and absent in *Plin2*^−/−^ adrenals. Two isoforms of Plin3 were detected, and both increased in *Plin2*^−/−^ adrenals compared with *Plin2*^+/+^. Different isoforms of Plin3 have been reported in human tissue (42) but not in mice. Within the Plin family, the Plin2 protein shares the highest homology to Plin3. In consistence with what has been reported in other cell types (43, 44), it seems as if the loss of the Plin2 protein is accounted for by increased levels of Plin3 protein. Protein expression of HSL, catalyzing cytosolic lipolysis of CE-rich LDs, was similar in *Plin2*^+/+^ and *Plin2*^−/−^ adrenals and unaffected by fasting. protein kinase A-mediated activation of HSL, as determined by relative serine-565 phosphorylated HSL (p565-HSL), was unchanged by removal of Plin2 or fasting, albeit a tendency for higher p-HSL/HSL ratio was observed in fasted *Plin2*^−/−^ adrenals.

Our analyses so far suggest that elevated CE storage in *Plin2*^−/−^ adrenals results from altered coating of the LD surface, potentially also influencing cholesterol metabolism or uptake. Adrenal cortical cells acquire cholesterol mainly via de novo cholesterol synthesis and lipoprotein uptake. Hence, we analyzed the expression of genes in these pathways. A minor reduction of *Scarb1* (Scavenger receptor class B type 1) mRNA in chow-fed *Plin2*^−/−^ mice and unaltered expression of *Ldlr* (LDL receptor) (Fig. 3E) did not indicate transcriptional changes driving altered adrenal cholesterol uptake. Expression of *Hmgcr* (3-hydroxy-3-methylglutaryl-CoA reductase) mRNA, encoding the rate-limiting enzyme in de novo cholesterol synthesis, was marginally increased in fed *Plin2*^−/−^ adrenals, suggesting that de novo cholesterol synthesis may not be increased, even if CE-LD levels are elevated in *Plin2*^−/−^ adrenals. Fed *Plin2*^−/−^ adrenals had weakly upregulated mRNAs of *Soat1* (the endoplasmic reticulum resident sterol O-acyltransferase 1) and *Pcyt1a* (choline-phosphate cytidylyltransferase A). Soat1 esterifies cholesterol to CE, whereas Pcyt1a synthesizes CDP-choline, the rate limiting step in PC synthesis. Although the enzymatic activity of Pcyt1a is primarily posttranslationally regulated, increased mRNA expression of these genes in Plin2^−/−^ adrenals suggests that enzymes may favor expansion of CE-rich LDs.

### *Plin2*^−/−^ mice have unchanged levels of circulating corticosterone

**Fig. 4:**
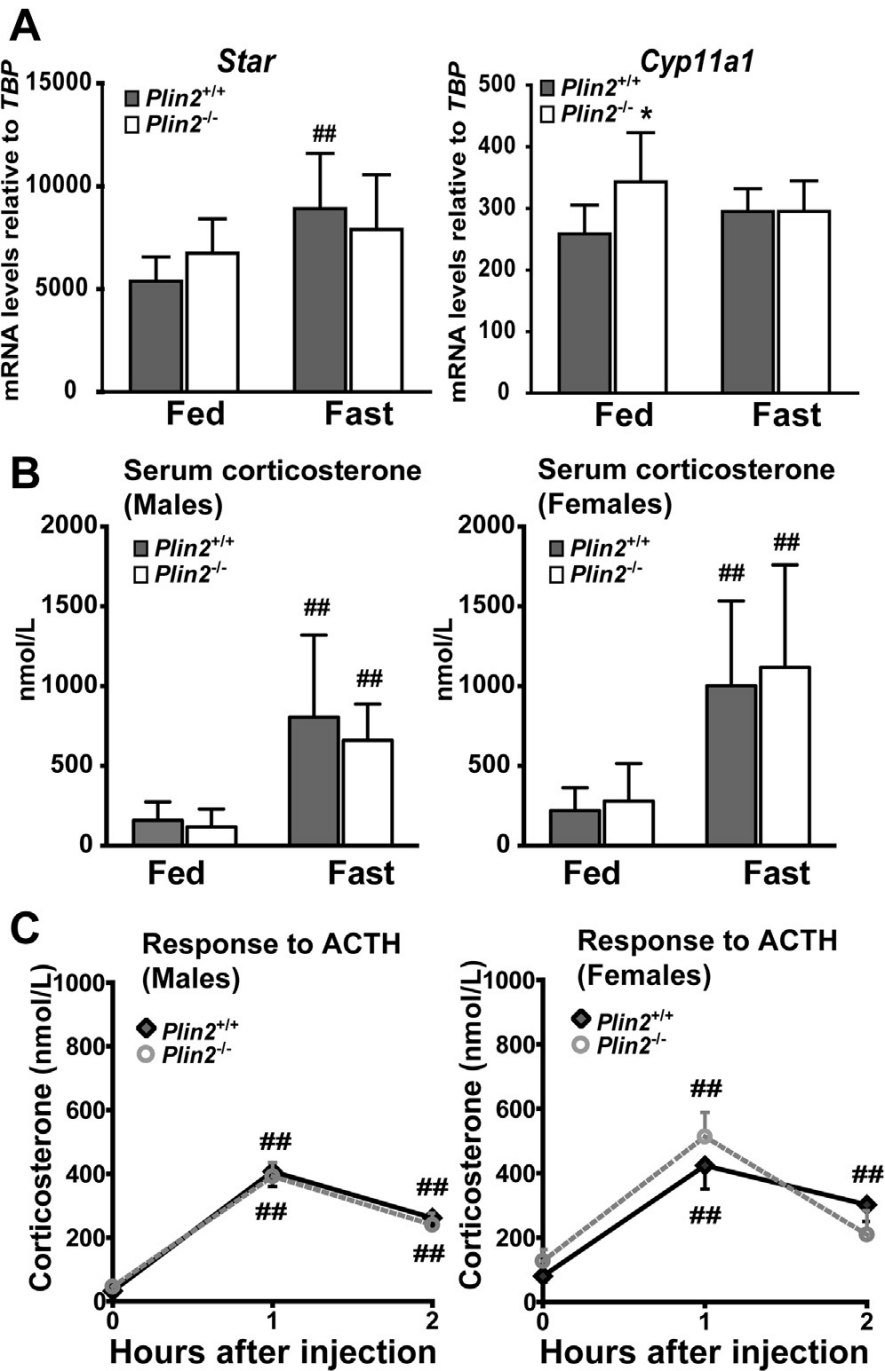
Serum corticosterone levels are unaltered in young *Plin2*^*+/+*^ and *Plin2*^*−/−*^ mice. Male and female 15-week-old *Plin2*^*+/+*^ and *Plin2*^*−/−*^ mice with ad libitum access to chow diet (Fed) or fasted for 24 h (Fast) were euthanized at 8–10 AM or subjected to ACTH stimulation. A: Expression of mRNAs for key enzymes in corticosterone synthesis (*Star* and *Cyp11a1*) relative to the expression of Tbp in fed or fasted female mice (n = 7–8). Results are presented as means ± SD (∗*P* < 0.05 indicates difference between *Plin2*^*+/+*^ and *Plin2*^*−/−*^ mice, ^##^*P* < 0.01 indicates difference between fed and fasted mice). B: Serum corticosterone levels in fed or fasted male and female *Plin2*^*+/+*^ and *Plin2*^*−/−*^ mice at 8–10 AM (n = 8). Results are presented as means ± SD (^##^*P* < 0.01 indicates difference between fed and fasted mice of the same genotype). C: Serum corticosterone levels in male and female *Plin2*^*+/+*^ and *Plin2*^*−/−*^ mice after ACTH stimulation. ACTH (i.p., 0.1 μg/g body weight) was administered to ad libitum fed mice at 9 AM. Blood samples collected before ATCH injection (baseline) and 1 and 2 h after injection were used to measure serum corticosterone. Results are presented as means ± SEM (males: n = 4; females: n = 6; ^##^*P* < 0.01 indicate differences from baseline). ACTH, adrenocorticotropic hormone; Cyp11a1, cytochrome P450 family 11 subfamily A member 1; Star, steroidogenic acute regulatory protein; Tbp, TATA-box binding protein.

In response to stress or energy depletion (fasting), the pituitary gland secretes ACTH that stimulates the adrenal cortex to mobilize CE, which is subsequently used by mitochondria to synthesize steroid hormones like corticosterone. Thus, we examined if elevated levels of adrenal CE in *Plin2*^−/−^ mice influenced steroid hormone production (steroidogenesis). First, we determined mRNA expression of *Star* (steroidogenic acute regulatory protein) and *Cyp11a1* (cytochrome P450 family 11 subfamily A polypeptide). The protein products of these two genes play essential roles during steroidogenesis: StAR transports cholesterol from the outer to the inner mitochondrial membrane, whereas Cyp11a1 catalyzes the conversion of cholesterol to pregnenolone, a rate-limiting step in steroid hormone synthesis (9). Expression of *Star* was slightly upregulated upon fasting but unaffected by removal of Plin2 (Fig. 4A), and only a minor increase in *Cyp11a1* mRNA was observed in the adrenals of chow-fed *Plin2*^−/−^ mice compared with *Plin2*^+/+^ mice.

We next measured circulating corticosterone in 15-week-old fed or fasted *Plin2*^+/+^ and *Plin2*^−/−^ mice. Although adrenal size and CE content were increased in *Plin2*^−/−^ adrenals, basal serum corticosterone levels were similar between chow-fed *Plin2*^+/+^ and *Plin2*^−/−^ mice (Fig. 4B). Fasting, which stimulates mobilization of CE-LDs, increased corticosterone levels about 3-fold compared with the fed state, independent of sex and genotype. We also measured corticosterone secretion in response to ACTH stimulation in chow-fed mice. Exogenous ACTH increased blood corticosterone 1 h after injection, whereas corticosterone levels declined toward basal levels 2 h after injection (Fig. 4C). No differences in corticosterone levels were observed between *Plin2*^+/+^ and *Plin2*^−/−^ mice injected with ACTH. To conclude, despite that *Plin2*^−/−^ adrenals had elevated precursor storage for steroidogenesis (increased levels of CE-rich LDs), steroid hormone synthesis seemed unaffected.

### Ceroid-like structures and multilamellar bodies accumulate in the adrenal cortex of *Plin2*^−/−^ mice

**Fig. 5:**
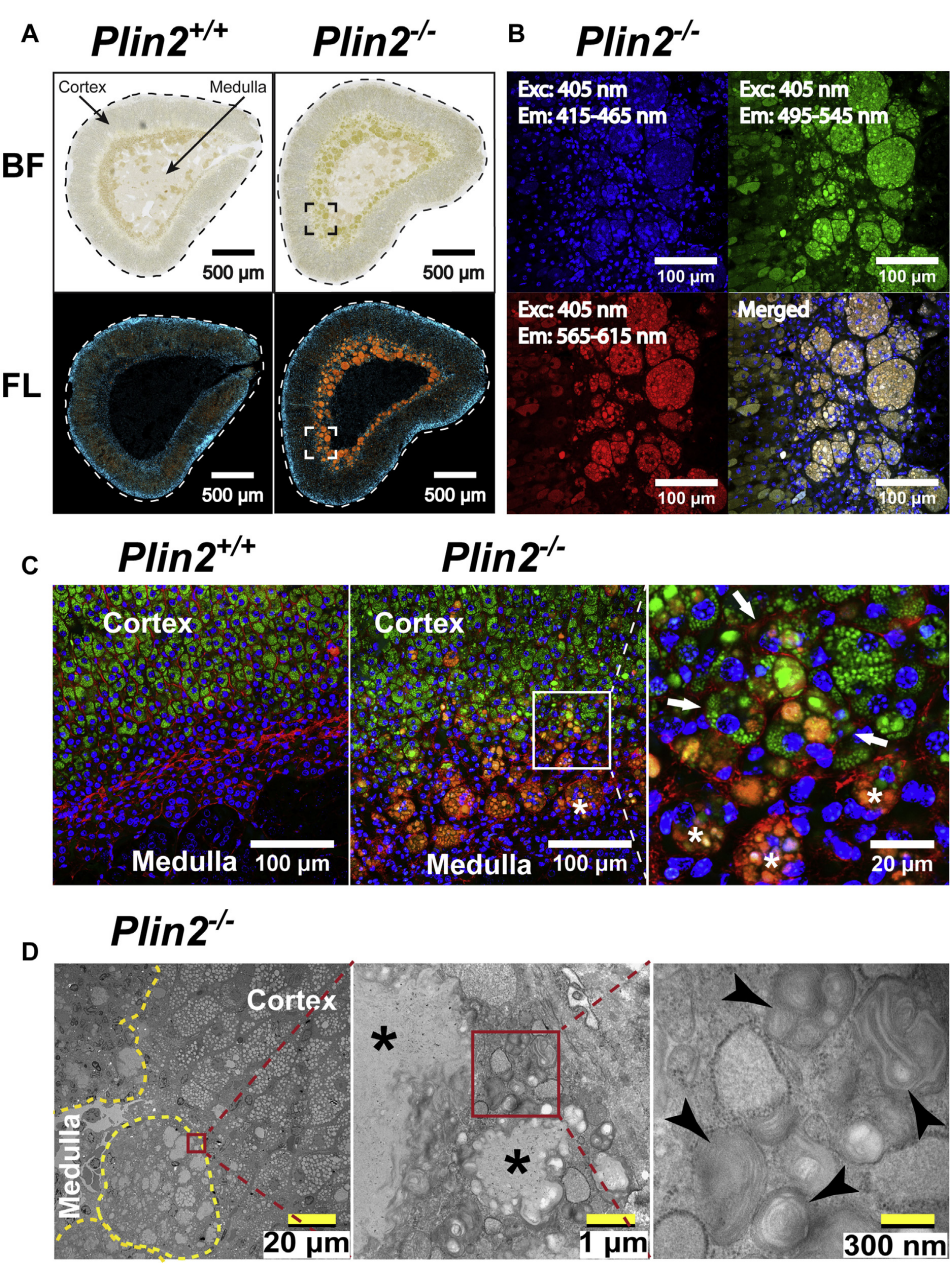
Ceroid-like structures and multilamellar bodies accumulate in the adrenal cortex-medulla border in *Plin2*^−/−^ mice. Adrenals from 30-week-old ad libitum fed female *Plin2*^+/+^ and *Plin2*^−/−^ mice were fixed and sectioned. Tissue sections were imaged with whole section scanning, confocal microscopy, or transmission electron microscopy (TEM). A: Whole section scans at the center planes of adrenals taken under bright field (BF) visualize accumulation of light brown-yellow aggregates in the basal adrenal cortex (close to medulla) in *Plin2*^−/−^ adrenals. The same sections were subsequently stained with Hoechst 33342 to visualize nuclei. Nuclei signals were exited at 330–375 nm and detected at 430–470 nm, whereas the autofluorescent aggregates (orange) were exited at 545–557 nm and visualized at 578–640 nm (FL). Scale bars are 500 μm. B: Confocal imaging of the above Hoechst 33342-stained sections (40× objective from the square marked in images in A). The granule-like aggregates in *Plin2*^−/−^ adrenals excited with a 405 nm laser have broad emission spectra (415–615 nm). Scale bars are 100 μm. C: Adrenal sections from *Plin2*^+/+^ and *Plin2*^−/−^ mice were stained with Hoechst 33342 to visualize nuclei (blue), Bodipy 493/503 to visualize lipid droplet (green), and CF568-Phalloidin to visualize F-actin in plasma membranes (red). High-resolution confocal imaging (63× objective, marked with a box) reveals multiple nuclei and vesicles (white asterisks) and deformed cortical cells containing lipid droplets (white arrows) relatively restricted to *Plin2*^−/−^ mice (scale bars 100 or 20 μm). D: Durcupan embedded ultra-thin sections from adrenals visualized with transmission electron microscopy. Examination of areas containing aggregates (encircled with yellow dotted lines) with higher resolution (red squared boxes indicate zoomed areas) reveals the presence of nonstructured lipid-like material (black asterisks) surrounded by multilamellar bodies (black arrow heads). Scale bars in images from left to right are 20 μm, 1 μm, and 300 nm, respectively. BF, bright field; Em, emission wavelength; Exc, excitation wavelength; FL, fluorescence.

By comparing morphologies of the adrenal cortex of *Plin2*^+/+^ and *Plin2*^−/−^ mice, we observed an abnormality at the inner layers close to the medulla in *Plin2*^−/−^ adrenals. In the cortex-medulla boundary of *Plin2*^−/−^ adrenals, clustered granule-like brownish-yellow structures were observed under bright field (Fig. 5A), with wide-spectral autofluorescence when excited by 405 nm light (Fig. 5B). High-resolution confocal microscopy revealed that these structures were located in close proximity to multiple nuclei and LDs surrounded by membrane compartments (Fig. 5C). These aggregates were identified as nonstructured membrane-enclosed lipid-like material surrounded by numerous multilamellar bodies when examined under higher magnification with transmission electron microscopy (Fig. 5D). Similar wide-spectral autofluorescent aggregates have been reported in the adrenals of a few genetically modified mouse models, where they have been described as “syncytial-lipoid structures” or “ceroid accumulation” (14, 45). The structures reported previously were also primarily observed in the cortex-medulla boundary, found to consist of multiple nuclei, lipid-like vesicles, and multilamellar bodies. In this article, these aggregates are described as “ceroid-like structures.”

### Ceroid-like structures accumulate in female *Plin2*^−/−^ adrenals with age

**Fig. 6:**
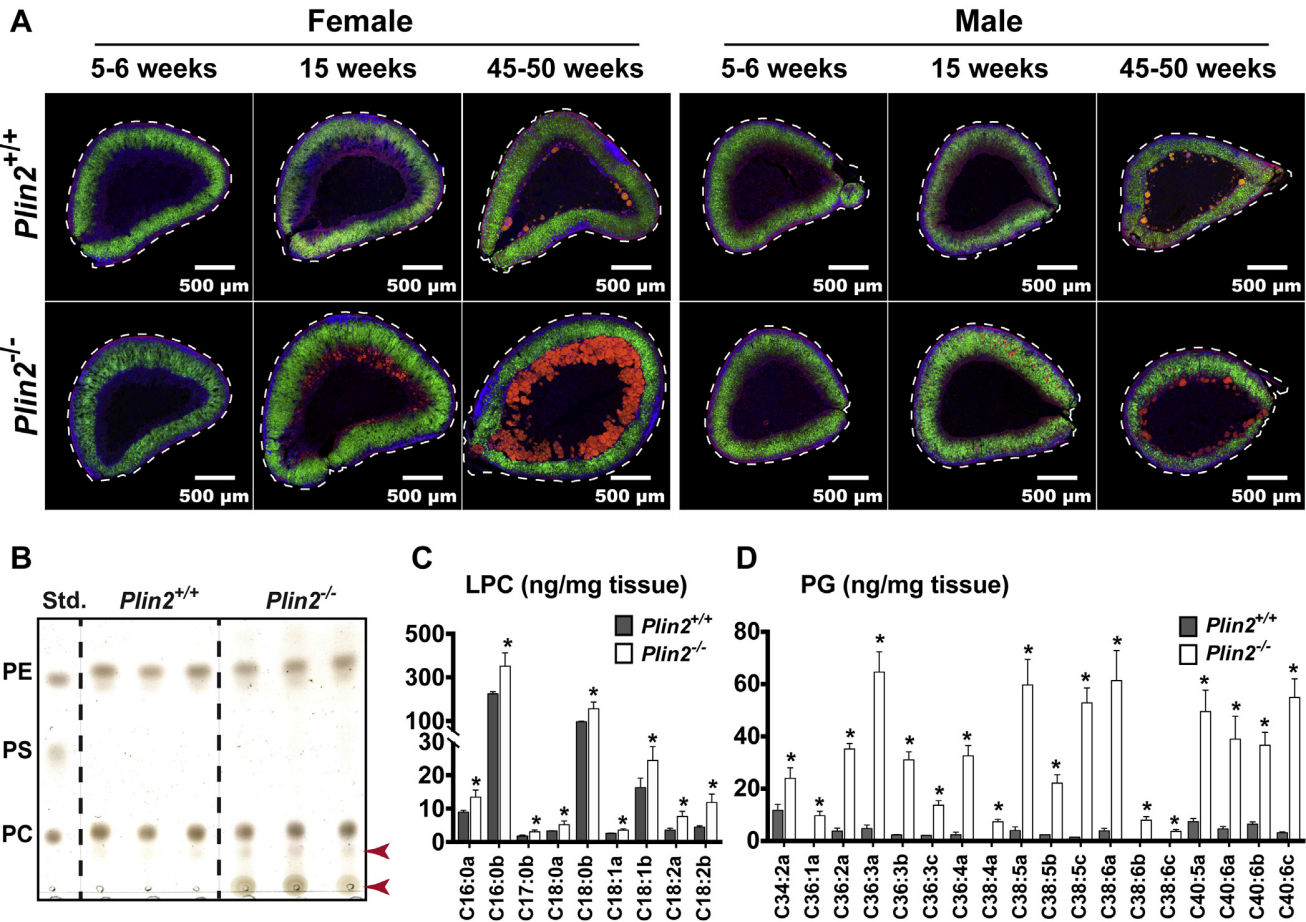
Accumulation of ceroid-like structures in adrenal glands is sex and age dependent. Adrenals were collected from 5-, 15-, and 50-week-old female and male *Plin2*^+/+^ and *Plin2*^−/−^ mice with ad libitum access to chow and used for histological or lipid analyses. A: Representative images of adrenals of female and male *Plin2*^+/+^ and *Plin2*^−/−^ mice sectioned through the center of the organ (n ≥ 3). Sections were stained with Hoechst 33342 to visualize nuclei (blue), Bodipy 493/503 to visualize lipid droplet (green), and CF568-Phalloidin to visualize plasma membranes (F-actin, red). The autoflourescent ceroid-like aggregates appear as orange/red structures. B: Phospholipids (PC, PS, and PE) in adrenals of 50-week-old female *Plin2*^+/+^ and *Plin2*^−/−^ mice analyzed with TLC. Extracted lipids normalized to 100 μg adrenal weight were applied per lane. PC-34:1, PS-34:1, and PE-34:1 were used as phospholipid standards. Following development of neutral lipids (not shown), a second developing phase was applied to separate polar phospholipids. The arrows indicate unidentified non/slow-migrating (highly polar) lipids present in *Plin2*^−/−^ but hardly visible in *Plin2*^+/+^ adrenals (n = 3). C: Lysophosphatidylcholine (LPC) species in adrenals of 50-week-old female *Plin2*^+/+^ and *Plin2*^−/−^ mice analyzed with HPLC-qTOF/MS. D: Phosphatidylglycerol (PG) species in adrenals of 50-week-old female *Plin2*^+/+^ and *Plin2*^−/−^ mice analyzed with HPLC-qTOF/MS. Results are presented as means ± SD (n = 3) relative to tissue weight. LPC, lysophosphatidylcholine; PC, phosphatidylcholine; PE, phosphatidylethanolamine; PG, phosphatidylglycerol; PS, phosphatidylserine.

To evaluate if the ceroid-like structures in adrenals of *Plin2*^−/−^ mice were influenced by sex or age, adrenal sections from 5-, 15-, or ∼50-week-old *Plin2*^+/+^ and *Plin2*^−/−^ mice were stained to visualize LDs (green), nuclei (blue), and the cell membrane (red). All adrenals were quite similar at 5 weeks of age, whereas sex and genotype differences were evident from 15 weeks of age (Fig. 6A). In 15-week-old *Plin2*^−/−^ females, ceroid-like structures accumulated in the cortex-medulla boundary. At 50 weeks of age, this accumulation was markedly aggravated with signs of adrenal cortical atrophy. In 15-week-old *Plin2*^−/−^ males, ceroid-like structures were less abundant compared with *Plin2*^−/−^ females and dispersed irregularly in the cortex layer. At 50 weeks of age, the accumulation was mostly in the cortex-medulla boundary and seemed slightly more abundant in *Plin2*^−/−^ males compared with *Plin2*^+/+^ males.

To characterize the lipid species that seemed to accumulate in the ceroid-like structures, lipids were extracted from the adrenals of 50-week-old female mice and separated by TLC. Neutral lipids were first removed with a nonpolar mobile phase. Then, the remaining polar lipids were separated with a second polar mobile phase. There was no obvious genotype difference in the content of PE and PC, the two major phospholipids found in adrenals (Fig. 6B). However, samples from *Plin2*^−/−^ adrenals contained bands hardly visible or absent in *Plin2*^+/+^ adrenals (Fig. 6B, red arrows), and especially large amounts of highly polar lipids that did not migrate on the TLC plate.

To reveal the identity of these highly polar lipids, we performed HPLC-qTOF/MS analyses on adrenals from 50-week-old female mice. Similar to what we found in 15-week-old mice (Supplemental Fig. S2), there were none or only minor differences in PC, PE, PS, phosphatidylinositol, sphingomyelin, and most ceramides (Cer) lipid species at 50 weeks of age (result not shown). In contrast, all detected LPC species were somewhat increased in *Plin2*^−/−^ adrenals compared with *Plin2*^+/+^ (Fig. 6C), whereas all PG species were massively increased (Fig. 6D). Of interest, PG species were only moderately increased at 15 weeks of age when the levels of these aggregates were much lower (Supplemental Fig. S2), implying that PG may be a component of the ceroid-like structures that accumulated with age in adrenals of female *Plin2*^−/−^ mice.

### Indications of altered autophagic flux and cholesterol efflux in *Plin2*^−/−^ adrenals

**Fig. 7:**
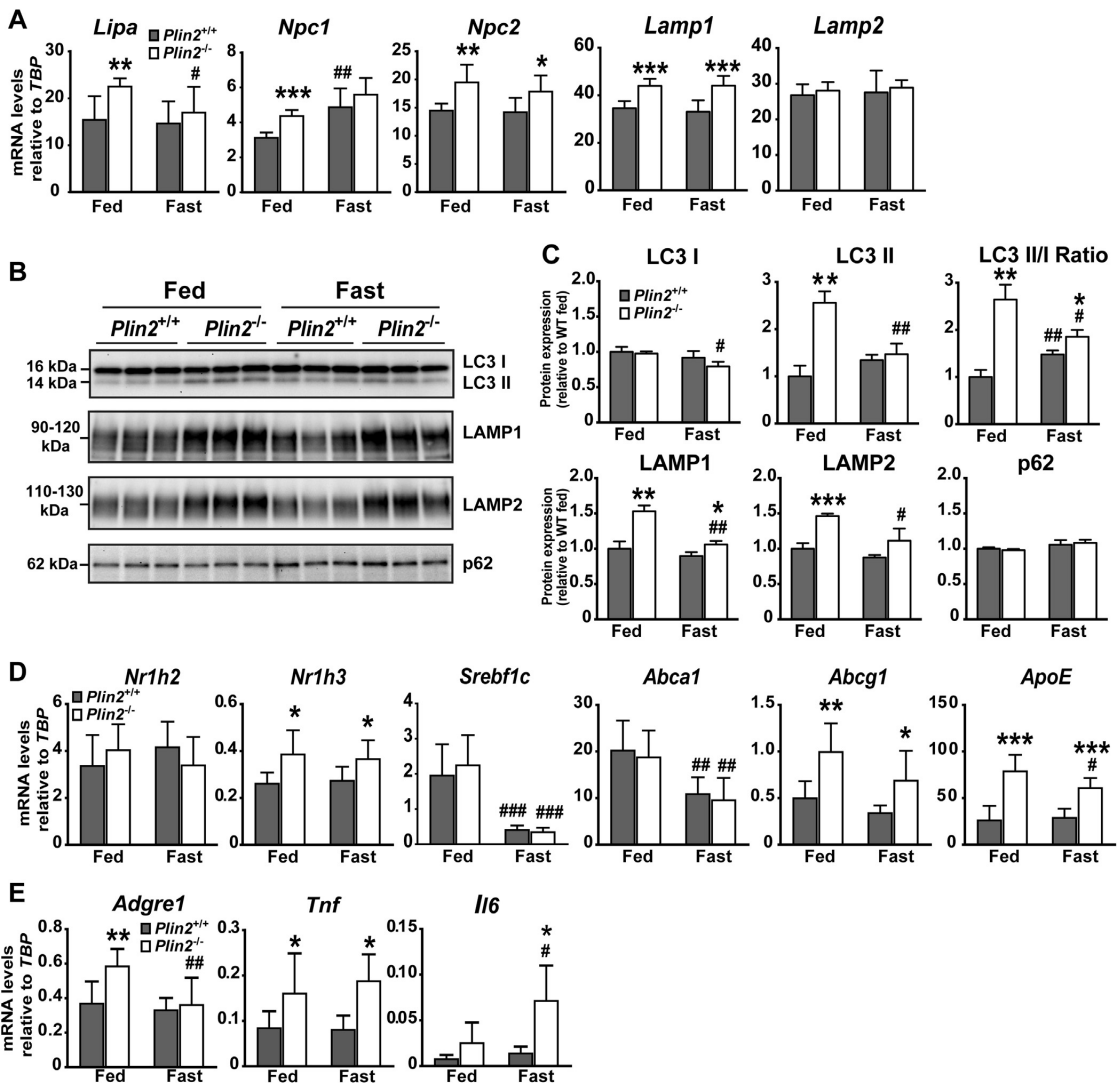
Expression of lysosomal, lipophagy, and cholesterol efflux-related genes in adrenals of *Plin2*^+/+^ and *Plin2*^−/−^ mice. Fifteen-week-old female *Plin2*^+/+^ and *Plin2*^−/−^ mice with ad libitum access to chow (Fed) or after 24 h of fasting (Fast) were euthanized at 8–10 AM. Adrenals were dissected free from the surrounding fat capsule prior to analyses. A: Expression of mRNAs encoding proteins associated with lysosomal lipid droplet degradation (*Lipa*), lysosomal cholesterol transport (*Npc1* and *Npc2*), and lysosomal membranes (*Lamp1* and *Lamp2*) relative to the expression of *Tbp* (n = 7–8). B: Protein content of cytosolic LC3 (LC3-I) and lipidated LC3 (LC3-II), lysosomal membrane proteins (Lamp1 and Lamp2), and the autophagy receptor (p62) in *Plin2*^*+/+*^ and *Plin2*^*−/−*^ adrenals. C: Quantification of immunoblot signals in B relative to levels measured for fed *Plin2*^+/+^ adrenals (n = 3). D: Expression of mRNAs encoding key transcription factors (*Nr1h2*, *Nr1h3*, and *Srebf1* isoform c) and transporters related to efflux of cholesterol (*Abca1*, *Abcg1*, *ApoE*) relative to the expression of *Tbp* (n = 7–8). E: Expression of genes related to macrophage infiltration and inflammation (*Adgre1*, *Tnf*, and *Il6*) relative to the expression of *Tbp* (n = 7–8). Gene expression data and quantified immunoblots are presented as means ± SD (∗*P* < 0.05, ∗∗*P* < 0.01, ∗∗∗*P* < 0.001 indicate difference between *Plin2*^+/+^ and *Plin2*^−/−^ mice; ^#^*P* < 0.05, ^##^*P* < 0.01, ^###^*P* < 0.001 indicate difference between fed and fasted mice of the same genotype). Abca1, ATP-binding cassette, subfamily A, member 1; Abcg1, ATP-binding cassette, subfamily G, member 1; Adgre1(F4/80, macrophage marker), adhesion G protein-coupled receptor E1; ApoE, apolipoprotein E; Lamp, lysosome-associated membrane protein; LC3, microtubule-associated protein 1A/1B-light chain 3; Lipa, lysosomal acid lipase A (LAL); Npc1/2, NPC intracellular cholesterol transporter 1/2; Nr1h2, nuclear receptor subfamily 1 group H member 2 (LXRβ); Nr1h3, nuclear receptor subfamily 1 group H member 3 (LXRα); p62, ubiquitin-binding protein p62; Srebf1c, sterol regulatory element binding transcription factor 1 isoform c (SREBP-1C); Tbp, TATA-box binding protein.

Multilamellar bodies are believed to originate from late-stage autolysosomes, and these tend to accumulate in tissues affected by lysosomal disorders (46). Moreover, elevated levels of PG seem to promote autophagy (47, 48). Based on these prior observations, we investigated if autophagy flux and lysosomes were altered in *Plin2*^−/−^ adrenals. Expression of mRNAs encoding proteins associated with lysosomal LD degradation, including lysosomal acid lipase (*Lipa*) and lysosomal membrane cholesterol transporters (*Npc1*, *Npc2*), were upregulated mainly in the adrenals of fed *Plin2*^−/−^ mice (Fig. 7A). *Lamp1* mRNA was increased in the adrenals of fed and fasted *Plin2*^−/−^ mice, whereas levels of *Lamp2* mRNA were unchanged. Still, protein expression of these lysosomal membrane markers was increased in the adrenals of fed *Plin2*^−/−^ mice but were nearly similarly expressed in fasted *Plin2*^+/+^ and *Plin2*^−/−^ mice (Fig. 7B, C). Protein levels of the cytosolic LC3-I isoform was essentially stably expressed, whereas the lipidated LC3-II isoform was specially elevated in the adrenals of fed *Plin2*^−/−^ mice. The autophagy receptor protein p62, which typically changed with altered autophagic flux (49, 50), was unaffected by genotype or fasting. These results suggest that lipidated LC3-II/lysosome aggregates accumulate in the adrenals of *Plin2*^−/−^ mice, especially in the fed state.

Altered lysosomal CE degradation and disturbed cholesterol transport to the cytoplasm may affect cellular cholesterol balance. We thus analyzed the expression of LXR-related transcription factors, which are important regulators of cellular cholesterol efflux (51, 52). Expression of LXRβ (*Nr1h2*) and *Srebf1c* mRNAs was unchanged by *Plin2* deletion, whereas LXRα (*Nr1h3*) mRNA and LXR-target genes involved in reverse cholesterol transport, such as *Abcg1* and *ApoE,* were upregulated in the adrenals of *Plin2*^−/−^ mice as compared with *Plin2*^+/+^ (Fig. 7D). The same genes are also typically expressed and upregulated in activated macrophages. Expression of mRNAs for the macrophage marker *Adgre1* and inflammatory markers such as *Tnfa* and *Il6* were inconsistently upregulated in the adrenals of *Plin2*^−/−^ mice (Fig. 7E). Overall, upregulation of certain cholesterol efflux-regulatory genes and macrophage inflammatory markers in *Plin2*^−/−^ adrenals suggest that the cholesterol efflux pathway is activated in cortical cells to reduce cholesterol levels and/or in macrophages to remove accumulating ceroid-like structures and damaged cells.

## Discussion

In most cell types, the dominant neutral lipid stored in LDs is TAG. Steroidogenic adrenal cortical cells are exceptions where LDs are almost exclusively filled with CE. Both classes of LDs are coated with Plin proteins, with Plin1-3 being detected in adrenals (22, 23, 53). The role of these Plins for CE storage and adrenal function have hitherto been unexplored. We now show that adrenal cortical cells lacking Plin2 have an increased content of CE-rich LDs and elevated basal levels of unesterified cholesterol, but without a substantial effect on steroidogenesis or secretion of corticosterone, at least in young mice. Moreover, *Plin2*^−/−^ adrenals had an increased protein content of Plin3, abnormal aggregation of ceroid-like structures consisting of multilamellar bodies, increased content of lipidated LC3 II and lysosomal membrane proteins, and massively increased levels of PG, indicative of autolysosome accumulation. The content of ceroid-like structures increased with age and appeared to be more abundant in female than in male mice. These results demonstrate that Plin2 is important for the regulation of CE-rich LDs and cellular cholesterol balance in the adrenal cortex.

The pool of cellular LDs is constantly balanced by the availability of lipids and hormonal stimuli that signal to promote LD growth or degradation. Plin proteins are important regulators of these processes, in particular lipolytic degradation (21, 54, 55). Previous studies demonstrate that Plin2 prevents TAG-rich LDs from degradation. Lack of Plin2 in mice reduces TAG-rich LDs in liver (55, 56, 57) and muscle (32), whereas Plin2 overexpression in various cell types tends to increase the content of TAG-rich LDs (57, 58, 59). In contrast, we observed enlarged and more numerous CE-rich LDs in *Plin2*^−/−^ adrenals. This discrepancy in the storage of neutral lipids in the absence of Plin2 may be cell type dependent or caused by distinct changes in the protein coating of TAG-rich versus CE-rich LDs. All mammalian cells examined to date express two or more Plin proteins at the LD surface, where removal of one Plin protein tends to change the relative abundance of the remaining Plin(s) at the LD surface (43, 60). We observed a reduction in Plin1a-c protein levels and an increase in Plin3 protein levels in *Plin2*^−/−^ adrenals. Plin1 protein is unstable and rapidly degraded when not bound to TAG-rich LDs (61). Although the same has not been firmly established for CE-rich LDs in steroidogenic cells, it is tempting to speculate that, with excess of Plin3, the LD surface available for docking of Plin1 might decrease, resulting in a net reduction in the Plin1a-c protein content. Such a shift in LD-coating may alter lipase recruitment, affecting CE storage, and disturb cholesterol balance.

Mouse models with disturbed adrenal cholesterol balance or altered mobilization of CE-rich LDs seem to affect steroidogenesis differently. LXRα^−/−^ mice have impaired cholesterol efflux resulting in increased levels of adrenal cholesterol and elevated levels of corticosterone in circulation (51). *Lipe*^−/−^ mice have a nearly diminished ability to mobilize CE-rich LDs by cytosolic lipolysis, resulting in increased CE storage but unaltered basal corticosterone levels and only moderately attenuated corticosterone secretion after ACTH stimulation (14, 15). We now demonstrate that *Plin2*^−/−^ mice, despite increased CE storage, have unaltered levels of plasma corticosterone in the basal state, after 24 h of fasting, or in response to exogenous ACTH stimulation. Together with a marked reduction in the LD content in both *Plin2*^+/+^ and *Plin2*^−/−^ adrenals after fasting, our results suggest that Plin2 is dispensable for mobilization of CE during steroidogenesis and that elevated levels of CE and unesterified cholesterol in adrenals do not necessarily influence steroidogenesis. Alternatively, the elevated levels of unesterified cholesterol observed in fed *Plin2*^−/−^ adrenals may not be accessible for mitochondrial steroidogenesis, e.g., by being sequestered in the accumulated autolysosomes and ceroid-like structures.

An unexpected structural change of *Plin2*^−/−^ adrenals is the abnormal accumulation of ceroid-like structures. These aggregates were especially abundant in old female *Plin2*^−/−^ mice, suggesting that these aggregates are formed in an age- and sex-dependent process. High-resolution confocal microscopy revealed the presence of premature ceroid-like structures in cortical cells containing LDs, suggesting their cortical cell origin. Cells enriched in these aggregates were often deformed with clusters of multiple nuclei and vesicles, an indication of controlled apoptotic cell death. Ultrastructural examination of the aggregates identified them as lipid-containing vesicles, often surrounded by numerous multilamellar bodies. Similar multilamellar bodies are found in late-stage autolysosomes, which tend to accumulate in tissues affected by lysosomal disorders (46), particularly in cases where cells accumulate cholesterol (62). The lipid composition of such ceroid-like structures containing multilamellar bodies is poorly characterized. Our lipid analyses revealed elevated levels of CE, LPC, and PG species in *Plin2*^−/−^ adrenals. CE accumulation in *Plin2*^−/−^ adrenals can most likely be explained by altered Plin coating of LDs and perhaps increased Soat1 expression resulting in increased cholesterol esterification. The mechanistic explanations for increased PG and LPC, however, is less clear. PG species were only modestly increased in young *Plin2*^−/−^ females but were increased massively in aged *Plin2*^−/−^ females that possess more pronounced ceroid-like structures. PG is a precursor to cardiolipins of the inner mitochondrial membrane (63). Unaltered steroidogenesis in *Plin2*^−/−^ adrenals indicates relatively intact mitochondrial function, arguing against a massive mitochondrial accumulation of PGs. PG has also been reported to increase during conditions of enhanced autophagic flux (47, 48) and is abundantly present in multilamellar bodies in surfactant-producing lipid-rich alveolar type II cells of the pulmonary alveolar (64, 65). Thus, our analyses suggest that PGs accumulate in *Plin2*^−/−^ adrenals as part of multilamellar bodies/ceroid-like structures and as a consequence of altered adrenal autophagic flux and/or reduced lysosomal function. Indeed, we did observe increased levels of lipidated LC3-II proteins and lysosomal membrane proteins in the adrenals of *Plin2*^−/−^ mice. LPC levels were relatively comparable in the adrenals of young and aged *Plin2*^−/−^ females and seemed elevated independent of ceroid-like structure abundance. LPC is produced by apoptotic cells and serves as a signal to attract phagocytes (66, 67). Thus, elevated LPC levels in *Plin2*^−/−^ females may be a consequence of activated apoptosis. It should be further noted that ceroid-like structures and multilamellar bodies also have been observed in the adrenals of *Lipe*^−/−^ mice (14, 15). Collectively, these observations indicate that deletion of either *Lipe* or *Plin2* leads to comparable effects on autolysosomes, lipophagy, and possibly apoptotic processes in adrenal cortical cells.

The increase in CE-rich LDs and unesterified cholesterol in *Plin2*^−/−^ adrenals suggests that both cholesterol flux and turnover of CE-rich LDs are affected by the lack of Plin2. Indeed, we found evidence for elevated transcriptional signaling resulting in upregulation of *PPARδ*, *Plins*, *LXRα*, *Abcg1*, and *ApoE* in *Plin2*^−/−^ adrenals. *Plins* are PPAR targets in several tissues and exhibit enhanced expression in response to elevated fatty acid levels and increased PPAR activity (37, 38, 39, 40). *Abcg1* and *ApoE* are known targets of LXRs. When intracellular cholesterol and cholesterol metabolites increase, LXRs are activated and stimulate transcription of genes important for cellular cholesterol efflux (68). These transcriptional changes support that cholesterol efflux is activated in *Plin2*^−/−^ adrenals, but additional studies will be needed to clarify if this pathway is activated in cortical cells, infiltrated macrophages, or both.

Our analyses suggest that impaired cytosolic CE hydrolysis results in an increased CE-rich LD content in the *Plin2*^−/−^ adrenals. In contrast, removal of Plin2 in most other cell types decreases TAG-rich LDs by enhancing lipolysis (32) and/or lipophagy (69). One exception is cardiomyocytes, where lack of Plin2 results in a minor increase in TAG-rich LDs, possibly due to reduced lipophagy (33). It is unclear to what extent the lipolytic and lipophagic pathways are affected in *Plin2*^−/−^ adrenals. Lipolytic degradation of adrenal CE-rich LDs is mainly catalyzed by the neutral lipase HSL (15) where HSL lipolytic activity is mainly regulated posttranslationally by its phosphorylation (18, 70) and binding to, e.g., Plin1 at the LD surface (71). In *Plin2*^−/−^ adrenals, HSL and phosphorylated HSL protein levels were unchanged, Plin1a-c proteins were reduced, whereas Plin3 levels were increased. It is presumable that such a shift in Plin protein coating of the LD surface may affect recruitment of HSL and thereby affect basal lipolytic CE degradation. Regarding lipophagy, the modest increase in the expression of lysosomal acid lipase (Lipa) and intracellular cholesterol transporters (Npc1 and Ncp2) (72) in *Plin2*^−/−^ adrenals suggests increased lysosomal CE degradation and increased release of unesterified cholesterol from autolysosomes to the cytosol. To supplement our in vivo studies, carefully designed studies using in vitro adrenal cortical cells lacking Plin2 will be required to dissect the exact mechanisms causing the imbalance in these delicately regulated processes. Our current data suggest that cytosolic lipolysis is reduced in *Plin2*^−/−^ adrenals, resulting in enlarged content of CE-rich LDs. This increase in CE stores seems partially compensated for by enhanced lipophagy, where bulk degradation results in elevated intracellular levels of unesterified cholesterol levels. With time, cholesterol and other components accumulate in autolysosomes and decompose them into ceroid-like structures, eventually resulting in cortical cell damage.

In summary, we demonstrate that *Plin2*^*−/−*^ adrenals have increased storage of CE-rich LDs and elevated cellular unesterified cholesterol levels, transcriptional activation of genes involved in cholesterol efflux, aggregation of ceroid-like structures, and accumulation of LPC and PG with age. Despite these changes, *Plin2* deletion does not significantly affect mobilization of CE-rich LDs required for corticosterone production. Our findings suggest that Plin2 is dispensable in providing cholesterol for steroidogenesis, most likely due to functional compensation through coating of CE-LD with Plin1c and/or Plin3. However, Plin2 does seem critical to regulate lipolytic or lipophagic degradation of CE-rich LDs in the basal state and is therefore important for balancing the flux of cellular cholesterol in adrenal cortex.

### Data availability

Data that support the findings of this study are available from the corresponding author upon reasonable request.

## Supplemental data

This article contains supplemental data.

## Conflict of interest

C. A. D. is a founder, shareholder, board member, and consultant in Vitas Ltd. T. B. is an employee and a shareholder of Vitas Ltd. All other authors declare that they have no conflicts of interest with the contents of this article.
