# Supplementary material for: Plin2 deletion increases cholesteryl ester lipid droplet content and disturbs cholesterol balance in adrenal cortex
Source: J Lipid Res. 2021 Feb 11;62:100048. doi: 10.1016/j.jlr.2021.100048 (PMC8044703; doi:10.1016/j.jlr.2021.100048)
Supplement: Supplemental Figures S1 & S2 and Tables S1 & S2 [file mmc1.docx]

**Supplemental Fig. S1**

**
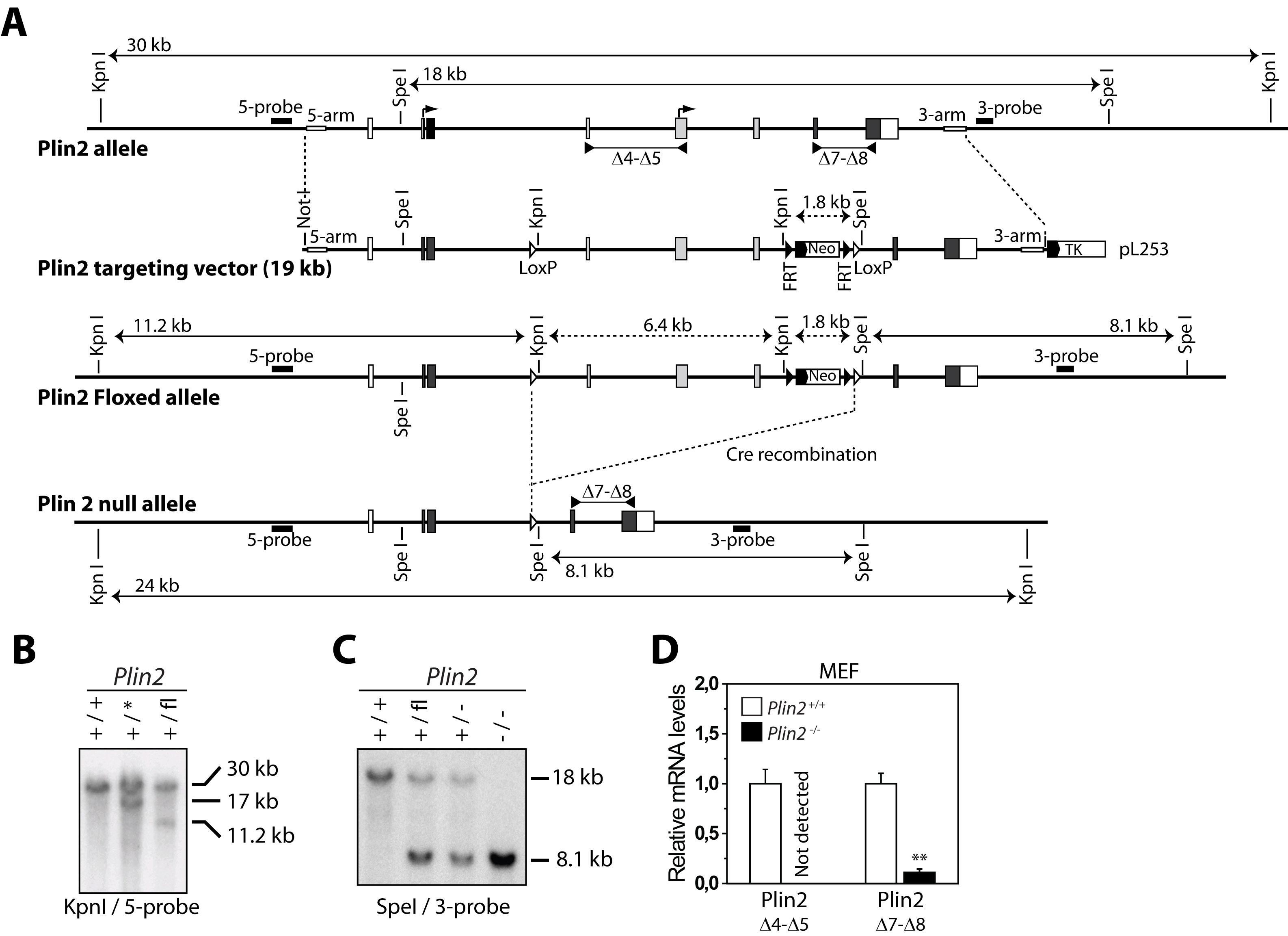
**

**Supplemental Fig. S1: Generation of the *Plin2* model**

A *Plin2* targeting vector with LoxP sites inserted in intron 3 and intron 6 was constructed using recombineering (see materials and methods). The floxed *Plin2* allele was introduced in embryonic stem (ES) cells using standard homologous recombination, followed by injection of positive ES cells into blastocysts for generation of mice containing a *Plin2* floxed allele. Prior to studies, *Plin2* floxed mice were crossed with MMTV-Cre recombinase mice to achieve global deletion of the *Plin2* allele (*Plin2*^-/-^ model). The *Plin2*^-/-^ model was backcrossed for >10 generations into C57BL/6NJr (Janvier Labs) prior to studies.

**A)** A schematic drawing of the *Plin2* allele (*Plin2*^+/+^), the *Plin2* targeting vector, the *Plin2* floxed allele and the *Plin2* null allele (*Plin2*^-/-^).

**B)** Southern blot analysis of DNA isolated from ES cells. The *KpnI* digested DNA was hybridized with the *Plin2* 5-probe resulting in the expected fragments of 30 kb (*Plin2*+ allele) and 11.2 kb (*Plin2*^fl^ allele).

A clone with insertion of the LoxP-Neo selection cassette in intron 6 but lacking insertion of the LoxP site in intron 3, expected to give rise to a ~17 kb fragment, is shown (*Plin2*^+/^*).

**C)** Southern blot analysis of DNA isolated from liver of *Plin2*^+/+^, *Plin2*^fl/+^, *Plin2*^+/-^, and *Plin2*^-/-^ mice. The *SpeI* digested DNA was hybridised with the *Plin2* 3-probe and confirmed to give the expected fragments of 18 kb (*Plin2*^+^ allele) or 8.1 kb (Plin2^fl^ or Plin2^-^ alleles).

**D)** Detection of *Plin2* mRNA transcripts in mouse embryonal fibroblasts (MEFs) isolated from *Plin2*^+/+^ and *Plin2*^-/-^ mice. The Δ4-Δ5 and Δ7-Δ8 primer pairs amplify across the exon4-5 or exon7-8 junctions, respectively.

**Supplemental Fig. S2**


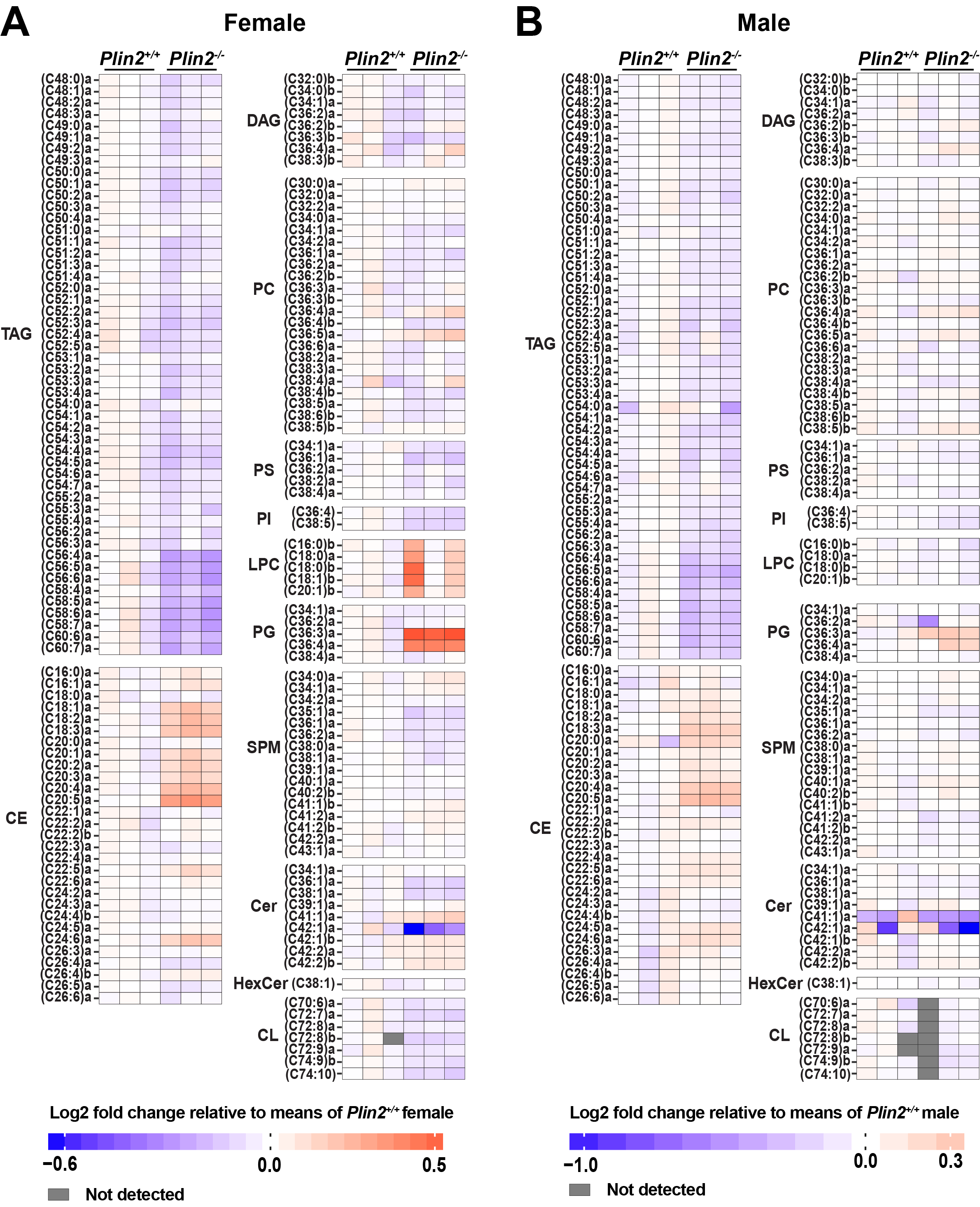


**Supplemental Fig. S2: Heat map of lipid species in *Plin2*^+/+^ and *Plin2*^-/-^ adrenals.**

Female and male *Plin2*^+/+^ and *Plin2*^-/-^ mice at 15 weeks of age with *ad libitum* access to chow were euthanized at 8-10 am. The surrounding fat capsule were removed under a stereo microscope prior to HPLC-qTOF/MS analysis.

**A)** Lipid species identified in adrenals of female *Plin2*^+/+^ (n=3) and *Plin2*^-/-^ (n=3) mice.

**B)** Lipid species identified in adrenals of male *Plin2*^+/+^ (n=3) and *Plin2*^-/-^ (n=3) mice.

Data are normalized against adrenal organ weight and presented as log2 fold change relative to means of lipid content in *Plin2*^+/+^ adrenals.

**Abbreviations**: CE, cholesteryl esters; Cer, ceramides; CL, cardiolipins; LPC, lysophosphatidylcholine; DAG, diacylglycerols; HexCer, hexosylceramides; PC, Phosphatidylcholine; PG, phosphatidylglycerol; PI, phosphatidylinositol; PS, Phosphatidylserine; SPM, sphingomyelin; TAG, triacylglycerols.

**Supplemental Table 1. Primers used for generation of the Plin2 Flox-Neo vector and identification of modified Plin2 alleles in ES cells and mice.**

| **Primers for generation of homology arms to extract the Plin2 genomic sequence** | | | | | |
| --- | --- | --- | --- | --- | --- |
| *Prime name* | | *Inserted RE site* | | *Sequence* | |
| Plin2_5-arm-fw | | NotI | | ATAAGCGGCCGCTAGCCTGCCTGGTTTGGTATTCA | |
| Plin2_5-arm-rev | | HindIII | | GTCAAGCTTGTGGAAGGTGCCTTTGGAGCTAT | |
| Plin2_3-arm-fw | | HindIII | | GCTAAGCTTAGACACACCCAAAACAAGGGTGA | |
| Plin2_3-arm-rev | | SpeI | | TCTACTAGTGTCAGGAGGATTGCTTGAGTCCA | |
| **Primers for generation of mini-vector targeting Plin2 intron 3** | | | | | |
| *Prime name* | | *Inserted RE site* | *Sequence* | | |
| Plin2-intron3_5-arm-fw | | NotI | ATAAGCGGCCGCAAAGCAATTGGAGGAGCAAAAGC | | |
| Plin2-intron3_5-arm-rev | | EcoRI | GTCGAATTCCTCAAAGGGTGATTCAGGGAGGT | | |
| Plin2-intron3_3-arm-fw | | BamHI, KpnI | ATAGGATCCTGGTACCCAGAATCTCGCAATCAAACACCA | | |
| Plin2-intron3_3-arm-rev | | SalI | GTCGTCGACGTGCTCTTAACCCTCCGTCACCT | | |
| **Primers for generation of mini-vector targeting Plin2 intron 6** | | | | | |
| *Prime name* | | *Inserted RE site* | *Sequence* | | |
| Plin2-intron6_5-arm-fw | | NotI | ATAAGCGGCCGCGCATGAACAGAGTGTGGTGCTGT | | |
| Plin2-intron6_5-arm-rev | | EcoRI, KpnI | GTCGAATTCTGGTACCGCTAGTAGATCAGGCTGGCCTCA | | |
| Plin2-intron6_3-arm-fw | | BamHI, SpeI | ATAGGATCCTACTAGTAAGTTCTATGGCAGCCAGGACAA | | |
| Plin2-intron6_3-arm-rev | | SalI | GTCGTCGACTATAATCCCAGGATTGGGGATGG | | |
| **Primers used to generate probes for Southern screening** | | | | | |
| *Prime name* | | *Sequence* | | | *Fragment size (bp)* |
| Plin2_5-probe-fw | | GGCATAGCTCACGCCAGTATTCA | | | 475 bp |
| Plin2_5-probe-rev | | TTACTCCCCTCCCCCACATTCTA | | |  |
| Plin2_3-probe-fw | | GCCATCTCTCCAGCCCTTTAGAA | | | 545 bp |
| Plin2_3-probe-rev | | CAATGTCCACACCTCTGCATCTG | | |  |
| **Primers used for PCR based genotyping** | | | | | |
| *Prime name* | *Sequence* | | | | *Obtained PCR fragment size (bp)* |
| Plin2 WT and Plin2-flox-Neo | | | | | |
| Plin2-scr1 | AGGCATGTGGAGGTGTTGGATT | | | | Plin2-WT: 275 bp |
| Plin2-scr2 | ATTGTTGGGGCTGGAGAGACAG | | | | Plin2-KO: 379 bp |
| Plin2-scr3 | ACACCCTCTGCTGGCTTCTGAG | | | | Plin2-WT: 359 bp |
| Plin2-scr4 | GCACAAACCTGCCCTACTTGATG | | | | Plin2-FRT-Neo-FRT cassette present: >2.2 kb |
| Plin2-scr5 | GTTGCCAGCCATCTGTTGTTTG | | | | Plin2-FRT-Neo-FRT cassette present: 516 bp |
| Plin2-scr4 | (see above) | | | | (Plin2-FRT sites recombined: no PCR product) |
| FRT sites recombined (floxed) | | | | | |
| Plin2-scr3 | (see above) | | | | Plin2- FRT-Neo-FRT cassette present:> 2.5 kb |
| Plin2-scr4 | (see above) | | | | Plin2-flox:477 bp |
| LoxP sites recombined (null allele) | | | | | |
| Plin2-scr1 | (see above) | | | | Plin2-WT: > XXX kb |
| Plin2-scr4 | (see above) | | | | Plin2-KO: 296 bp |
| Cre-fw | ACCAGGTTCGTTCACTCATGGAA | | | |  |
| Cre-rev | CAGACCAGGCCAGGTATCTCTGA | | | |  |
| Flp-fw | CGTGGCCAGGACAACGTATACTC | | | |  |
| Flp-rev | GCTGCCACTCCTCAATTGGATTA | | | |  |

**Supplemental Table 2. Primers used for RT-qPCR**

| Gene name | Accession | Forward primer | Reverse primer | Product size | Intron length |
| --- | --- | --- | --- | --- | --- |
| Plin1 | NM_001113471.1 | ACCTGGAGGAAAAGATCCCG | TTCGAAGGCGGGTAGAGATG | 87 | 1316 |
| Plin2 | NM_007408.3 | GGGCTAGACAGGATGGAGGA | CACATCCTTCGCCCCAGTTA | 99 | 2215 |
| Plin3 | NM_025836.3 | CGAAGCTCAAGCTGCTATGG | TCACCATCCCATACGTGGAAC | 98 | 1147 |
| Plin4 | NM_020568.3 | ACCAACTCACAGATGGCAGG | AGGCATCTTCACTGCTGGTC | 109 | 1213 |
| Plin5 | NM_001077348.1 | GGTGAAGACACCACCCTAGC | CCACCACTCGATTCACCACA | 115 | 568 |
| Lipe | NM_001039507.2 | TCACGCTACACAAAGGCTGC | GAGAGTCTGCAGGAACGGC | 83 | 2590 |
| PPARa | NM_001113418.1 | ACTACGGAGTTCACGCATGT | GTCGTACACCAGCTTCAGCC | 74 | 1710 |
| PPARg | NM_001127330.1 | TTGCTGTGGGGATGTCTCAC | AACAGCTTCTCCTTCTCGGC | 70 | 12003 |
| PPARd | NM_011145.3 | ACATGGAATGTCGGGTGTGC | CGAGCTTCATGCGGATTGTC | 108 | 1590 |
| Nr1h3 | NM_001177730.1 | GACTTCAGTTACAACCGGGAAGA | ATTCATGGCTCTGGAGAACTCAAA | 90 | 5129 |
| Nr1h2 | NM_001285517.1 | GAAGGCGTCCACCATTGAG | AAGTCGTCCTTGCTGTAGGT | 108 | 473 |
| Tbp | NM_013684.3 | AGCCTTCCACCTTATGCTCAG | GCCGTAAGGCATCATTGGACT | 90 | 1145 |
| Cd36 | NM_001159557.1 | AGGCATTCTCATGCCAGTCG | TGTACACAGTGGTGCCTGTT | 119 | 8174 |
| Scarb1 | NM_001205083.1 | ATGCAGCTGAGCCTCTACATC | CACAGCAACGGCAGAACTAC | 83 | 2626 |
| Pcyt1a | NM_001163160.1 | CTACAGAGGGGCTACACTGC | ATCAACTCGTTCTTGCAAGTGG | 81 | 2830 |
| Mc2R | NM_001301372.1 | ACCACAAATGATTCTGCTGCTTC | TCACGATTGTTTCTGCAGTATGTT | 84 | 1490 |
| Star | NM_011485.5 | GCCTTGGGCATACTCAACAAC | TAGCACTTCGTCCCCGTTC | 72 | 1010 |
| Cyp11a1 | NM_001346787.1 | CAAGAACATCCAGGCCAACATT | TACCTTCAAGTTGTGTGCCATCT | 106 | 4211 |
| Srebf1c | XM_006532716.2 | GGAGCCATGGATTGCACATTT | CAGCATAGGGGGCGTCAAA | 91 | 3078 |
| Srebf2 | NM_033218.1 | TGACTCTCGGGGACATCGAC | CACCTCCAGGGAAGGAGCTA | 105 | 22247 |
| Tnf | NM_013693.3 | CCACCACGCTCTTCTGTCTAC | CTGATGAGAGGGAGGCCATT | 85 | 516 |
| Il6 | NM_031168.2 | TGATGGATGCTACCAAACTGGA | GGTACTCCAGAAGACCAGAGG | 74 | 3059 |
| Abca1 | NM_013454.3 | ACCGAGGAAGAAGCTCGATG | GGTCGGGAGATGAGATGTGG | 103 | 11328 |
| Abcg1 | NM_009593.2 | AAGGTCTCCAATCTCGTGCC | CCCTGATGCCACTTCCATGA | 96 | 2131 |
| Ldlr | NM_010700.3 | GACTGCAAGGACATGAGCGA | TGTCCAAGCTGATGCACTCC | 103 | 1860 |
| Soat1 | NM_009230.3 | CTGGGGAGAATCCTGAGCAAG | CCAACAGCCTCTTCTTGGCTA | 105 | 8676 |
| Adgre1 | XM_006523601.1 | TGTACGTGCAACTCAGGACT | TCCTGGAGCACTCATCCACA | 94 | 1365 |
| ApoE | NM_009696.4 | CCTGAACCGCTTCTGGGATT | CCATCAGTGCCGTCAGTTCT | 107 | 413 |
| Hmgcr | NM_001360165.1 | GCCTTGTGATTGGAGTTGGC | ACACTGACATGCAGCCGAAG | 78 | 2804 |
| Lipa | NM_001111100.1 | CCGCTACTTCAATTGGGACG | CACTTCAGCATCGCACTCTG | 92 | 5359 |
| Npc1 | NM_008720.2 | GCCTGGGTACATTTGGAGGA | GCGGTGGCATTGTTGTAGTTC | 82 | 3433 |
| Npc2 | NM_023409.4 | ACTGCCCCATCCAGAAAGAC | TCAAGTTTCCATTCCACCACCA | 100 | 3507 |
